# Microbiome of vineyard soils is shaped by geography and management

**DOI:** 10.1186/s40168-019-0758-7

**Published:** 2019-11-08

**Authors:** Emanuela Coller, Alessandro Cestaro, Roberto Zanzotti, Daniela Bertoldi, Massimo Pindo, Simone Larger, Davide Albanese, Enzo Mescalchin, Claudio Donati

**Affiliations:** 1 Faculty of Science and Technology, Free University of Bozen, P.za Università 1, 39100 Bolzano, Italy; 2 Unit of Computational Biology, Research and Innovation Centre, Fondazione Edmund Mach, Via Mach 1, 38010 San Michele all’Adige, Italy; 3 Technology Transfer Center, Fondazione Edmund Mach, Via Mach 1, 38010 San Michele all’Adige, Italy

## Abstract

**Background:**

Despite their importance as a reservoir of biodiversity, the factors shaping soil microbial communities and the extent by which these are impacted by cultivation are still poorly understood. Using 16S rRNA gene and ITS sequencing, we characterized the soil microbiota of vineyards and of neighboring permanent grassland soils in the Italian province of Trentino, and correlated their structure and composition to location, chemical properties of the soil, and land management.

**Results:**

Bacterial communities had a core of conserved taxa accounting for more than 60% of the reads of each sample, that was influenced both by geography and cultivation. The core fungal microbiota was much smaller and dominated by geography alone. Cultivation altered the structure and composition of the soil microbiota both for bacteria and fungi, with site-specific effects on their diversity. The diversity of bacterial and fungal communities was generally inversely correlated across locations. We identified several taxa that were impacted by the chemical properties and texture of the soil.

**Conclusions:**

Our results highlight the different responses of bacterial and fungal communities to environmental factors and highlight the need to characterize both components of the soil microbiota to fully understand the factors that drive their variability.

## Background

The microorganisms that colonize soil are amongst the most abundant and diverse life forms on Earth, contributing to all geochemical processes on a global scale [1], and constituting a rich source of yet uncharacterized natural products of potential interest for pharmaceutical or biotechnological applications [2]. The biodiversity of soil microbial communities is increasingly recognized as a major factor for human health both directly, by limiting the spread of potential pathogens, and indirectly, by contributing to processes that provide clean air, water, and healthy food [3]. Soil serves as a primary reservoir for plant-colonizing bacteria [4], that play a major role in determining plant productivity [5] and preventing invasion by bacterial pathogens [6].

Bacteria, archaea, and fungi are the dominant components of soil microbiota, generally accounting for more than 99% of the microbial biomass in soil samples [7]. Large-scale surveys have shown that the diversity encompassed by soil microbial communities exceeds what is found in host-associated communities [8, 9], probably as a consequence of the enormous range of environmental conditions that can be experienced by microorganisms in surface soils [7, 10]. Bacterial communities are characterized by pronounced heterogeneity at small spatial scales, and by a more homogeneous structure over large spatial scales [10], showing biogeographical patterns that are significantly weaker than what is found for plants [11]. Besides bacteria, fungi are the other major component of the soil microbiota, playing crucial roles both as saprotrophs, plant mutualists, and pathogens [8] and competing with bacteria for access to nutrients through the production of antimicrobial compounds [12]. Large-scale studies have shown that a large fraction of fungal taxa found in soils is not represented in sequence databases, that the diversity of soil fungal communities is influenced by a variety of climatic and edaphic factors [13, 14], and that soil fungal communities exhibit evident patterns of geographical clustering [8].

Understanding how agricultural practices impact the soil microbiota is an important subject towards a more sustainable agriculture. Recent studies on deforested lands have shown that land use has long-term effects on soil microbiota structure and diversity [15]. Both parameters are consistently altered by high levels of nutrient inputs related to human activities [16]. However, it is only by comparing cultivated and non-cultivated soils across locations, that it is possible to quantify the relative weights of differences related to location compared to these related to cultivation. In this work, we have characterized the bacterial and fungal microbiota in soils collected in 10 sites from Trentino, a region in the Italian Alps, with the aim of defining the taxonomic structure of both the bacterial and fungal components of the soil microbiota, and study the relative effects of location, chemical characteristics of the soil, and land use. To this purpose, sites were chosen to have cultivated patches (vineyards) surrounded by permanent grasslands. In each site, samples were collected from the vineyards and from grasslands at different distances from the vineyards. By comparing cultivated and permanent grasslands from the same site, we identified the species that were consistently impacted by cultivation.

It has recently been shown that a relatively small number of ubiquitous species dominate the global soil microbiota [17], while, at the other end of the spectrum, rare taxa play an important role as a reservoir of biological functions and resiliency against environmental changes [18]. However, how the set of ubiquitous species changes from global to local scale, and if geographical factors or land use can significantly alter its size is not known. Moreover, despite their importance as a component of the soil microbiota, no data are available concerning the existence of a core of shared fungal species in soil samples and how this core depends on soil type and is impacted by human intervention. If similar general ecological mechanisms drive the establishment of bacterial and fungal microbiota in soil, we should expect that the size of the core of bacterial and fungal microbiota has the same dependency on location and land use. On the other hand, differences in the way land use or location impact the core bacterial and fungal microbiomes could highlight fundamental differences in the factors that drive the colonization of soil by these two classes of microorganisms.

Defining the species that characterize the soil of a given area and how these are influenced by external factors is especially relevant in the case of grapevine, since soil has been suggested to be the major source of grapevine-associated microbiota [4]. Although the existence of a connection between soil and grape microbiota is still debated [19], the correlation between grape microbiota and wine metabolite profiles has suggested that microbial communities contribute to define the regional characteristics of wine [20, 21], leading to the hypothesis of the existence of a “microbial terroir” of wine grapes [21]. Here, we defined the core of ubiquitous bacterial and fungal species that were present in all soil samples and tested if its size was altered by location or type of cultivation. We found that while the bacterial component of the microbiome had a core of conserved species that accounted for more than 60% of the sequenced reads, and that was shaped both by location and land use, the core fungal microbiome was smaller and determined by geographic factors that dominated differences due to land management.

Finally, having characterized fungal and bacterial communities in the same samples allowed us to highlight the effects of their interactions. Recently, a global study of the variability of the topsoil microbiome has provided evidence of a strong antagonism between the two communities [12] mediated by antibiotic production by the soil fungi. Here, by correlating fungal and bacterial diversity in the same samples, we observed patterns consistent with the hypothesis that complex fungal communities impose a strong selection on bacterial community causing a loss of diversity in the latter, as expected in the case of antibiotic-mediated interactions [22].

## Materials and methods

### Sample collection

The sampling sites were identified in 10 vineyards from 4 different locations (Ala, Besagno, Mori and S. Felice) because of their contiguity, at least along 20 m, to perennial crop-covered surfaces (Additional file 1: Figure S1). The experimental protocol set 3 samplings points respectively between the rows (V) and in the perennial crop area at a distance of 8 (P1) and 16 (P2) meters from the border of the vineyard (see Additional file 2: Figure S2). The choice for 8 and 16 m guaranteed the reproducibility of sampling in each location, since the grassland areas surrounding vineyards have different and relatively small size. In all the sampling spots, grasses were mainly constituted from species of *Poaceae* family. In the vineyard sites (V), no predominant species were found while in the two grasslands (P1 and P2), the dominant species were *Arrhenatherum elatius, Bromus erectus*, and *Trisetum flavescens.* For each position 6 equally spaced repetitions were performed, for a total of 180 samples. Additional file 14: Table S1 shows site localization, sampling dates, and technical characteristics of the vineyards (planting year, previous crop). All samples had a similar range of soil texture (loam, sandy clay loam, sandy loam, and silty loam). Vineyards soils (V) were statistically different from permanent grassland (both P1 and P2) for lower quantity of soil organic matter (SOM) (50.63 ± 1.43 g/kg, 70.17 ± 2.32 g/kg, and 73.04 ± 2.26 g/kg respectively) and total nitrogen (N tot) (2.78 ± 0.07 g/kg (V), 3.92 ± 0.14 g/kg (P1), 4.07 ± 0.13 g/kg (P2)), instead they had higher concentrations of total carbonate (CaCO3) (199.73 ± 14.82 g/kg (V), 171.35 ± 15.01 g/kg (P1), 156.33 ± 14.67 g/kg (P2)) and available heavy metals (Cu DTPA 51.23 ± 3.94 mg/kg (V), 16.9 ± 1.71 mg/kg (P1), 6.66 ± 0.46 mg/kg (P2), and Zn DTPA 10.53 ± 0.94 mg/kg (V), 7.7 ± 0.60 mg/kg (P1), 6.21 ± 0.57 mg/kg (P2)). See Additional file 3: Figure S3.

Samplings were executed collecting 20 cm of soil by means of a manual, one-piece, 7-cm-diameter drill for loamy soils (Eijkelkamp, Edelman model). For chemical analysis and for taxonomic purposes of bulk soil, the first 5 cm of soil were removed. Each sample consisted of 4 drillings that were homogenized in a signed, plastic bag. From every one of them, a small volume of soil was collected in a 50-ml tube and chilled to 6/8 °C during the sampling time after which they were frozen at − 18 °C.

### DNA extraction, library preparation, and sequencing

The soil samples were freeze-dried and sieved with a 0.2-mm-mesh size and stored at − 80 °C until DNA extraction. Total DNA was extracted from 0.25 g of each composite soil sample using the PowerSoil DNA isolation kit (MO BIO Laboratories Inc., CA, USA) according to the manufacturer’s instructions. Total genomic DNA was amplified using primers specific to either the bacterial and archaeal 16S rRNA gene or the fungal ITS1 region. The specific bacterial primer set 515F (5′-GTGYCAGCMGCCGCGGTAA-3′) and the 806R (5′-GGACTACNVGGGTWTCTAAT-3′) was used [23] with degenerate bases suggested by Apprill et al .[24] and by Parada et al .[25]. Although no approach based on PCR amplification is free from bias, this primer pair has been shown to guarantee good coverage of known bacterial and archaeal taxa [26]. For the identification of fungi, the internal transcribed spacer 1 (ITS1) was amplified using the primer ITS1F (5′-CTTGGTCATTTAGAGGAAGTAA-3′) [27] and ITS2 (5′-GCTGCGTTCTTCATCGATGC-3′) [28]. All the primers included the specific overhang Illumina adapters for the amplicon library construction.

For the 16S V4 region, each sample was amplified by PCR using 25 μl reaction with 1 μM of each primer. More in detail 12.5 μl of 2× KAPA HiFi HotStart ReadyMix and 10 μl forward and reverse primers, were used in combination with 2.5 μl of template DNA (5–20 ng/μl). PCR reactions were executed by GeneAmp PCR System 9700 (Thermo Fisher Scientific) and the following cycling conditions: initial denaturation step at 95 °C for 5 min (1 cycle); 28 cycles at 95 °C for 30 s, 55 °C for 30 s, 72 °C for 30 s; final extension step at 72 °C for 5 min (1 cycle).

For the ITS1 region, each sample was amplified by PCR using 25 μl reaction with 10 μM of each primer. More in detail 22 μl of premix FastStart High Fidelity PCR System (Roche) and 2 μl forward and reverse primers were used in combination with 1 μl of template DNA (5–20 ng/ul). PCR reactions were executed by GeneAmp PCR System 9700 (Thermo Fisher Scientific) and the following cycling conditions: initial denaturation step at 95 °C for 3 min (1 cycle); 30 cycles at 95 °C for 20 s, 50 °C for 45 s, 72 °C for 90 s; final extension step at 72 °C for 10 min (1 cycle).

The amplification products were checked on 1.5% agarose gel and purified using the Agencourt AMPure XP system (Beckman Coulter, Brea, CA, USA), following the manufacturer’s instructions. Afterward, a second PCR was used to apply dual indices and Illumina sequencing adapters Nextera XT Index Primer (Illumina), by 7 cycles PCR (16S Metagenomic Sequencing Library Preparation, Illumina). The amplicon libraries were purified using Agencourt AMPure XP system (Beckman), and the quality control was performed on a Typestation 2200 platform (Agilent Technologies, Santa Clara, CA, USA). Finally, all barcoded libraries were pooled in an equimolar way and sequenced on an Illumina® MiSeq (PE300) platform (MiSeq Control Software 2.5.0.5 and Real-Time Analysis software 1.18.54.0).

### Bioinformatic processing of the sequences

The sequences were assigned to samples using sample-specific barcodes and saved in FASTQ-formatted files. Sequences were deposited to the European Nucleotide Archive (ENA) with study accession PRJEB31356. Raw data FASTQ files were analyzed using the software pipeline MICCA [29] v. 1.6.1 (Microbial Community Analysis).

Raw overlapping 16S paired-end reads were assembled (merged) using the procedure described in [30]. Paired-end reads with an overlap length smaller than 200 bp and with more than 50 mismatches were discarded. After trimming forward and reverse primers, merged reads shorter than 240 bp and with an expected error rate higher than 0.5% were removed.

Reads with less than 60% similarity to the sequences present in the Greengenes [31] database (clustered at 85%, release 13_5) were discarded using VSEARCH [32] v2.3.4. Filtered sequences were clustered into operational taxonomic units (OTUs) at 97% identity using the denovo greedy algorithm available in MICCA. OTUs were taxonomically classified using the Ribosomal Database Project (RDP) Classifier [33] v2.11. Multiple sequence alignment (MSA) was performed on the denoised reads applying the Nearest Alignment Space Termination [29, 34] (NAST) algorithm and the phylogenetic tree was inferred using FastTree [35] (v2.1.8).

Raw overlapping ITS paired-end reads were merged, and merged sequences with an overlap length smaller than 100 bp and with more than 32 mismatches were discarded. After primer trimming, merged reads shorter than 150 bp and with an expected error rate higher than 0.5% were removed. Filtered sequences were clustered at 97% identity using the denovo greedy algorithm and OTUs were taxonomically classified using the RDP Classifier v2.11 and the UNITE database (release 07/04/2014) [36]. Sequences that were not in the UNITE database were indicated as “unclassified” while sequences that are present, but without taxonomic information available, were classified as “fungi_unidentified” plus a numeric suffix for the taxonomic level. For instance, in the case of the family level, sequences that have no taxonomic information other than being fungi were labeled as “fungi_ unidentified _1_1”.

To compensate for different sequencing depths, samples were rarefied to an even depth of 16,000 reads for 16S and 22,000 for ITS sequences.

### Statistical analysis of the data

Biom files were imported into R v3.4.3 using the *phyloseq* package [37] v1.22.3 for downstream statistical analysis. Alpha diversity was calculated using the Shannon entropy [38]. Beta-diversity was calculated using the Bray-Curtis distance. Permutational MANOVA (PERMANOVA) statistical tests were performed using the R package *vegan* v2.4–6 with the *adonis2()* function with 999 permutations. Taxa significantly different between vineyard soils and permanent grasslands were identified using the generalized linear models implemented in the R package *DESeq2* [39] v1.18.1 on the unrarified reads, filtered selecting OTUs that were represented by at least 10 reads in more than 25% of the samples to increase the reproducibility of the results [40]. Random Forest models to identify relevant environmental factors to predict alpha-diversity were built using the *randomForest* [41] v4.6–14 R package. Linear models of the alpha-diversity as a function of the environmental variables were built using the *lm* function in R, and contrasts between different locations and soil types were determined using the function *contrast* from the R package *contrast* v0.21. Multilevel hierarchical linear models were built using the *lmer* function of the *lme4* v1.1–17 R package, modeling slope as fixed effect and intercept as a random variable. *p* values were estimated using the package *lmerTest* 3.0–1. Correlation between taxa at all taxonomic levels and environmental variables were identified using the *MICtools* software package [42], a recent software package that identifies significant correlations between large datasets using an approach based on mutual information. Briefly, significant correlations were identified calculating the total information coefficient estimator TIC_e_ [43]. The associated (FDR corrected) *p* values were estimated using a permutation-based strategy. The strength of the association was then quantified using the MIC_e_ estimator of the maximal information coefficient [43] and the Spearman’s rank correlation coefficient ρ. Cladograms of the taxonomy were drawn using the R package *Metacoder* [44]. The size of the nodes was proportional to the relative abundance of the taxa while the color represented Spearman’s rank correlation coefficient ρ.

### Texture and chemical characteristics of the soil

All samples were air-dried at room temperature and sieved < 2 mm. For the analysis of organic carbon and total inorganic carbon, 50 g of sieved soil were ground using an agate-ball mill (PM 4000, Retsch GMBH, Haan, Germany) to reduce the particle size < 0.02 mm.

Soil texture was determined as the percentage of sand (2.0–0.050 mm), silt (0.005–0.002 mm) and clay (< 0.002 mm) by wet sieving and with the use of hydrometer after dispersion with sodium hexametaphosphate.

Soil pH was measured in aqueous solution suspension (ratio 1: 2.5, soil: water) using an INOLAB LEVEL 2 (WTW, Weilheim, Germany) pH-meter equipped with a SenTixTM41 pH Electrode (WTW, Weilheim, Germany).

Total inorganic carbon was determined by volumetric method with a Dietrich-Fruehling calcimeter by measuring the CO2 evolved after HCl treatment of soil according to ISO 10693 (ISO 10693:1995. Soil quality–determination of carbonate content–Volumetric method), whereas active lime was quantified by titration after reaction of soil with ammonium oxalate 0.1 M (Drouineau method). Both were expressed as g CaCO3/kg of soil.

Total C and N were measured after dry combustion in excess oxygen using a CN analyzer (MacroVario, Elementar, Langenselbold, Germany). Two hundred milligrams of milled soil were weighted in tin foil and analyzed following manufacturer’s instructions. Organic C was then calculated as the difference between total C and total inorganic C as reported by ISO 10694 (ISO 10694:19959. Soil quality–determination of organic and total carbon after dry combustion]. The C/N ratio was calculated as the ratio between organic C and total N.

The available fractions of Cu, Zn, Pb, and Cd were extracted with a DTPA 0.005 M, CaCl2 0.01 M, and triethanolamine 0.1 M solution and measured with an ICPOES spectrometer (Optima 8300, Perkin Elmer, Waltham, USA) equipped with a cyclonic nebulizer, using the following wavelengths: Cu = 324.752 nm, Zn = 213.857 nm, Pb = 220.353 nm, Cd = 226.502 nm. For the quantification, the instrument was calibrated using a certified standard solution (Merck, Darmstadt, Germany).

## Results

Soil samples were collected from 10 sites in the Trentino province (Additional file 1: Figure S1 and Additional file 14: Table S1a). Each site was characterized by sampling the vineyard (V) and the grasslands at 8 (P1) and 16 m (P2) from the grapevine row (see “Methods” section). For each triplet, 6 samples were collected, for a total number of 180 samples. After discarding 3 samples due to the low quantity of extracted DNA, a total of 5,705,432 amplicon sequences from the V4 region of 16S rRNA gene and 7,350,959 sequences from the ITS region were obtained from the remaining 177 samples. The 16S and ITS sequences were clustered into 21,113 and 12,542 operational taxonomic units (OTUs, 97% identity), respectively. After dropping one 16S sample due to the low number of reads, 16S and ITS samples were evenly rarefied to 16,000 and 22,000 reads per sample, respectively. After rarefaction, the dataset was composed of 19,584 bacterial and 12,101 fungal OTUs.

### Microbiota composition is conditioned by cultivation for both bacterial and fungal communities

**Fig. 1:**
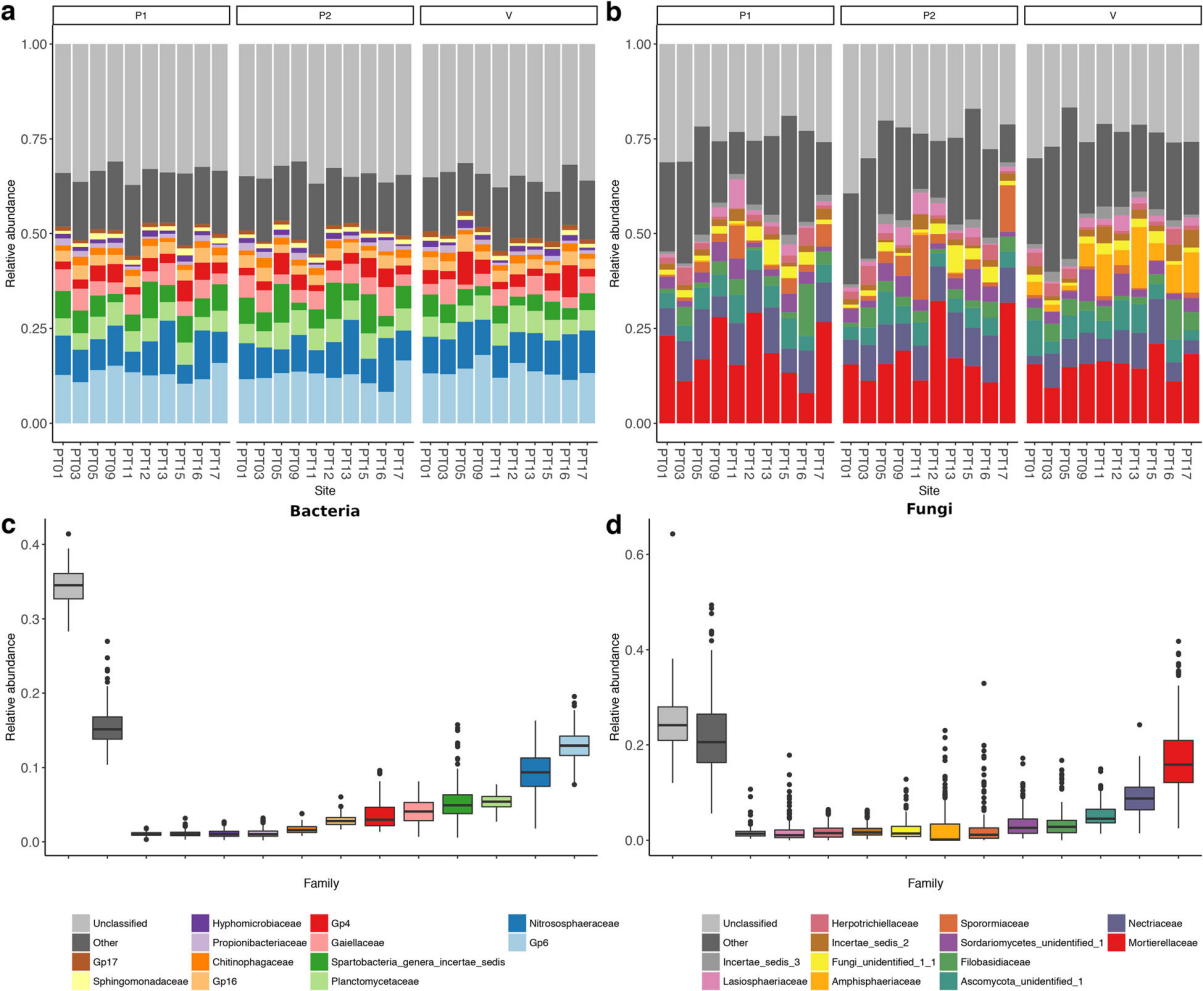
Taxonomic structure of the soil bacterial (**a**) and fungal (**b**) microbiota at the family level. Only the 12 families with the largest mean relative abundance are shown. **c**, **d** Box and whiskers plot of the 12 families with largest mean relative abundance across all samples. Families other than the top 12 were classified as “other”

The dominant bacterial Phyla were *Acidobacteria* (22.7%), *Proteobacteria* (18.8%), and *Actinobacteria* (16.5%), while on average, 14.1% of the reads could not be classified. At the family level (Fig. 1a, c), the fraction of bacterial OTUs that could not be classified grew to 34%, while for those that were classified the dominant family was *Gp6* (13%), followed by *Nitrosospheraceae* and *Planctomycetaceae* (9% and 5%, respectively). For fungi, the dominant Phyla were *Ascomycota* (51.8%), *Zygomycota* (20.1%), and *Basidiomycota* (11.2%), while 12.1% was constituted by unclassified OTUs. At the family level (Fig. 1b, d), the dominant taxa were *Mortierellaceae* (17.4%) followed by *Nectriaceae* (8.8%) and a family of unidentified *Ascomycota* (5.2%). The fraction of fungal OTUs not classified at the family level was 25%.

To identify taxa that were significantly impacted by cultivation, we modeled the read counts using generalized linear models [39] (GLMs) taking baseline differences between the sites into account. For this analysis, samples from both types of permanent grassland (both P1 and P2) were considered together. For bacteria, we found 336 OTUs that were significantly differentially abundant (*p* < 0.01, FDR corrected) between samples from vineyard soils and from permanent grasslands from the same site. Of these, 224 were higher in vineyard samples and 112 in grasslands (Additional file 15: Table S2). The 10 most significant ones included taxa from the families *Gp4, GP6*, Hyphomicrobiaceae, and Planctomycetaceae (Additional file 4: Figure S4)*.* For fungi, we found 57 taxa significantly (*p* < 0.01, FDR corrected) more abundant in the vineyard than permanent grassland soil from the same site, and 37 taxa significantly less abundant in vineyard samples (Additional file 16: Table S3). Amongst these, the most significant were taxa from the family *Amphisphaeriaceae*, that in vineyard samples accounted for up to 20% of the fungal microbiota (Additional file 5: Figure S5). Other differently distributed taxa included unidentified *Pleosporales*, unidentified *Ascomycota*, *Hyaloscyphaceae*, and *Sordariomycetes*.

### Bacterial communities have a core of conserved species that is shaped by both location and land use

**Fig. 2:**
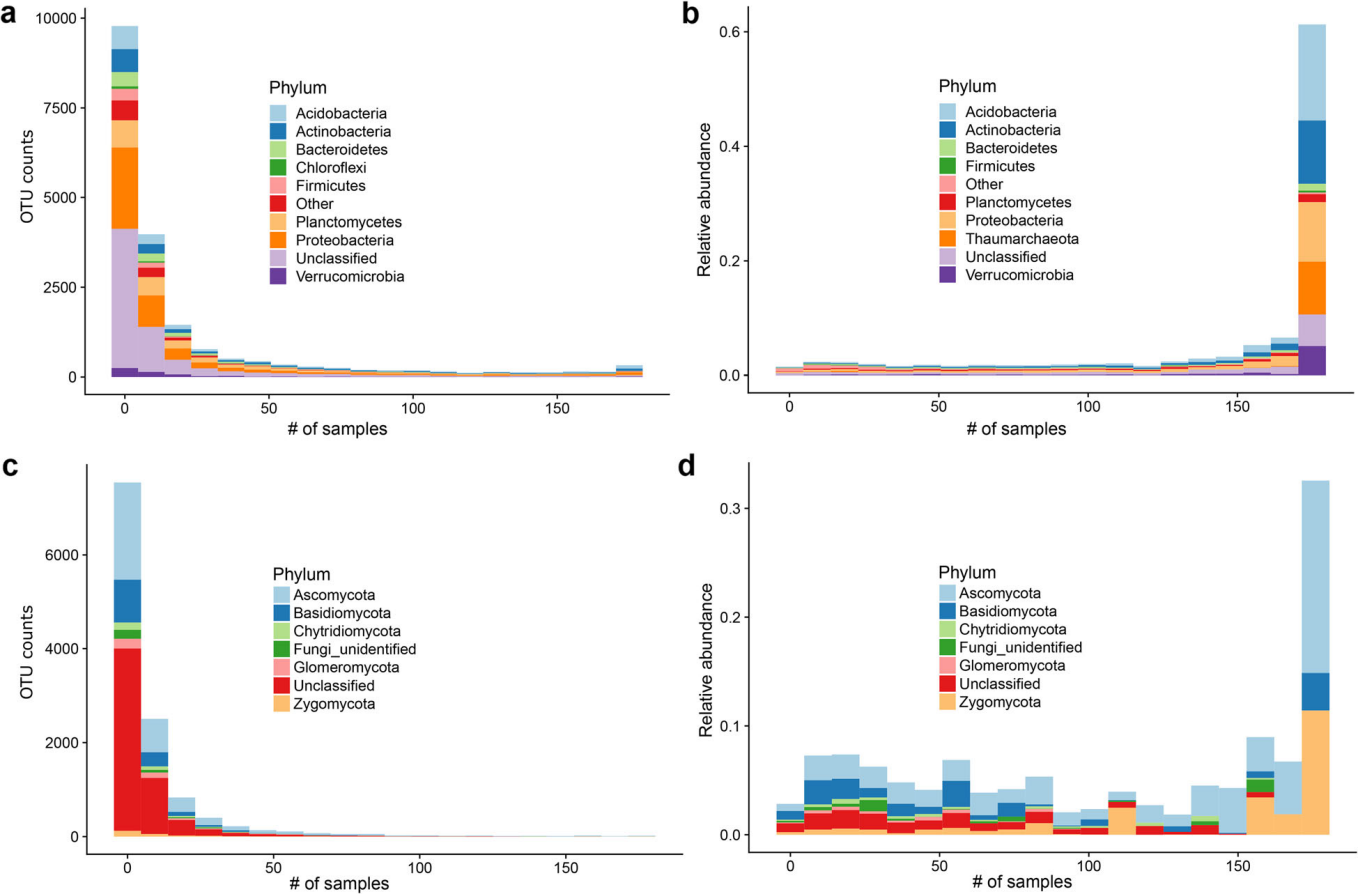
Core and specific bacterial and fungal microbiome. **a** Distribution of the number of bacterial OTUs that are specific to a given number of samples, classified at the *Phylum* level. Only the eight more representative taxa are shown. The remaining reads are either “unclassified” or annotated as “other”. **b** Total relative abundance of bacterial OTUs that are specific to a given number of samples. **c** distribution of the number of fungal OTUs that are specific to a given number of samples, classified at the *Phylum* level. Only the seven more representative taxa are shown. The remaining reads are either “unclassified” or annotated as “other”. **d** Total relative abundance of fungal OTUs that are specific to a given number of samples

The distribution of OTUs across samples (Fig. 2a) showed that the largest fraction of OTUs was specific to a small number of samples while only a small set of OTUs was ubiquitous. Specifically, out of 19,584 OTUs, only 162 were present in all samples, 484 were present in at least 80% of the samples, and 961 in at least 50% of the samples.

Despite the relatively low number, the core OTUs accounted on average for 48 ± 3.8% of the bacterial reads of each sample. Using a more relaxed definition of the core OTUs, i.e., defining as “core” those OTUs that are present in more than 95% of the samples, core OTUs accounted on average for 64 ± 3% of each microbiome (Fig. 2b). For comparison, the specific OTUs (i.e., those present in less than 5% of the samples) that are the vast majority of the bacterial OTUs, only accounted for an average of 1.5 ± 0.5% of each bacterial microbiome. *Acidobacteria*, a minority component of the specific microbiome, was the dominant component of the core microbiome. Other frequent components of the core microbiome included *Actinobacteria* and *Proteobacteria*, while *Bacteroidetes* and *Firmicutes* were almost absent.

We next asked if the size of the core microbiome was influenced by the cultivation type or geographical origin of the samples. We found that the core microbiome of P1, P2, and V samples included 250, 256, and 240 OTUs, respectively, while the site-specific core microbiome varied between 372 and 638 OTUs (Additional file 17: Table S4). In both cases, the core microbiome was significantly larger than expected by random sampling (Wilcoxon rank-sum test, *p* values 1.16 × 10^−6^ for site and 7.8 × 10^− 3^ for cultivation type, respectively), suggesting that both these factors favor the colonization of soil by a defined set of bacterial species.

In general, in any given dataset, the number of core OTUs (core microbiome) decreases with the number of samples, since each newly added sample might miss OTUs that were core in the reduced dataset. Therefore, it is possible to estimate the true size of the core microbiome, i.e., the number of OTUs that are always present in this kind of soils independently of the sampling size by extrapolating from a random subsampling. The results are shown in Additional file 6: Figure S6a, where we plot the number of core OTUs as a function of the number of samples. The curve can be fitted by a power-law decay converging to a plateau of 117 ± 3.5 OTUs that is the estimated size of the core bacterial microbiome of these soils.

### The core of conserved fungal species is small and determined by location, but not land use

Compared to bacteria and archaea, the fungal component of the soil microbiome (the mycobiome) was more variable across the different sampling sites. Indeed, out of 12,101 total OTUs, only 5 were present in all samples (Fig. 2c). Core OTUs accounted on average for 15 ± 5% of each sample (35 ± 8% for OTUs present in more than 95% of the samples, Fig. 2d), while the specific OTUs (i.e., those present in less than 5% of the samples) represented an average of 4.8 ± 0.3% of each mycobiome (Fig. 2d). From a taxonomic point of view, the core mycobiome was dominated by *Ascomycota*, *Zygomycota*, and a small fraction of *Basidiomycota*. Using the same subsampling strategy outlined above, the size of the core mycobiome as a function of the number of samples was again well described by a power-law (Additional file 6: Figure S6b) that in this case converged to a value close to zero (4.1 ± 0.4 OTUs). Differently to what found for bacteria, the core of conserved fungal species in the three cultivation types (17, 10, and 13 OTUs for V, P1, and P2 samples, respectively) was not significantly larger than expected by random sampling (*p* value 0.58). On the contrary, the different sites had a site-specific core mycobiome (Additional file 17: Table S4) significantly larger than expected by random sampling (*p* value 3.2 × 10^−4^). These results suggest that geographical factors dominate the composition of the soil mycobiota that show a higher level of variability compared to bacteria, and that cultivation is not able to select a defined set of fungal species across different locations overcoming the differences due to geographical factors.

### Bacterial and fungal richness vary with site and cultivation, and are correlated in a site-specific manner

**Fig. 3:**
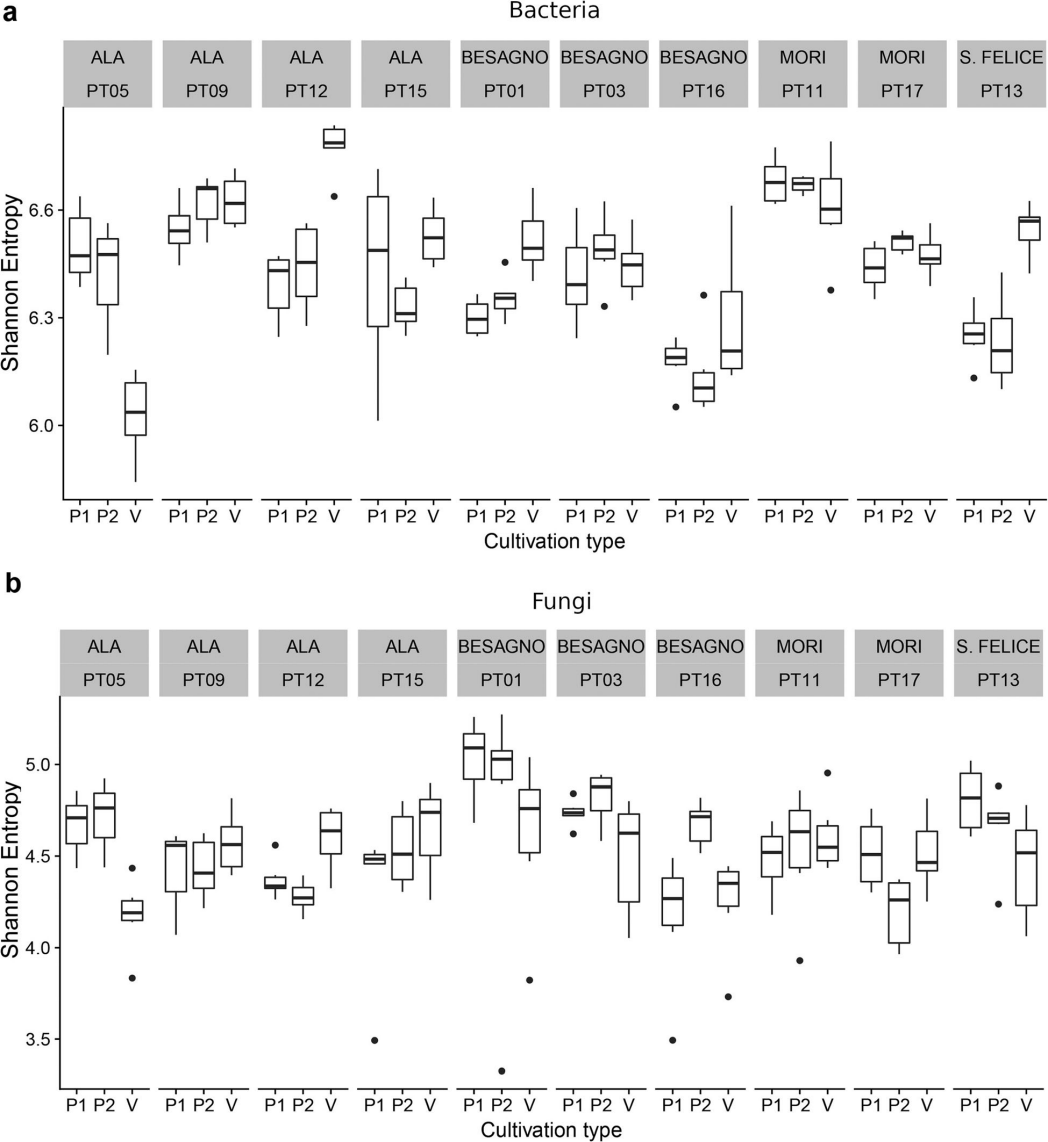
Shannon entropy in the different sampling sites. **a** Bacteria. **b** fungi

The microbiome richness, or α-diversity, varied widely across locations, sites, and cultivation type (Additional file 18: Table S5 and Additional file 19: Table S6) for both bacteria and fungi (Fig. 3a, b respectively). We first tested the differences across the locations (Additional file 18: Table S5) and sampling sites (Additional file 19: Table S6) finding that in many cases the differences were significant. We then tested whether the microbiota richness was significantly different between the three cultures within the same sampling site. Despite the fact that samples from the vineyards had often a bacterial α-diversity significantly different from surrounding permanent grassland, we could not highlight an unambiguous effect of cultivation across all sites (Additional file 20: Table S7). For bacteria, in no case the P1 and P2 samples had significantly different richness (exceeding the 0.05 significance level), while in three cases the V samples have a significantly higher, and in one lower richness than the corresponding P1 samples (Additional file 20: Table S7). For fungi, in two cases, the α-diversity of the P2 samples were significantly different from the P1 samples, while in three cases, the V samples had a lower α-diversity than the corresponding P1 samples, and in one case higher (Additional file 20: Table S7).

To highlight the possible effects of competition between bacteria and fungi, we tested by linear modeling whether there was a correlation between the bacterial and fungal α-diversities in the same samples. We found that the correlation across all samples was negligible (adjusted *R*^2^ = -0.006, *p* value = 0.86, Additional file 21: Table S8). However, correlating the diversity indexes within each site the correlation was significant (adjusted *R*^2^ = 0.22, *p* value = 2.22*10^−6^, Additional file 22: Table S9). In most sites, the fungal α-diversity was negatively correlated with the bacterial α-diversity, with the exception of PT05 and PT12 both from the same area (Ala), where we found a positive correlation between bacterial and fungal α-diversity (*R*^2^ = 0.51, slope 0.91, *p* value = 0.0016, Additional file 23: Table S10 and *R*^2^ = 0.48, slope 0.64, *p* value = 0.0008, Additional file 24: Table S11, respectively). For the other sites, using a linear mixed-effect model where the intercept was treated as random variable, we obtained an estimated value of − 0.59 (*p* value = 0.002, Additional file 25: Table S12) for the slope of the correlation between the fungal and bacterial α-diversity.

### Chemical characteristics of the soil partially explained the variability in richness

To identify soil features that have an impact on α-diversity, we built a random forest model including the texture (percentage of sand, silt, and clay) and chemical characteristics of the soil (absolute quantity of CaCO_3_, Cu, Zn, Pb, Cd, soil organic matter, carbon-to-nitrogen ratio-C/N -, and pH). The model was able to account for 58.25% of the α-diversity variability for bacteria and archaea. The more relevant characteristics were the absolute quantity of CaCO_3_, the percentage of sand, and the percentage of silt, followed by the absolute quantities of Zn and Cu, and by pH. Differently from what recently found in global surveys [7, 12, 45], the effect of pH was moderate, probably due to the relatively small range of pH values sampled in the present study. By linear modeling, we found that the bacterial microbiome richness was significantly positively correlated (Additional file 7: Figure S7 and Additional file 26: Table S13) with CaCO_3_ and silt and negatively with sand, Cu, and Zn. The correlation with pH was not statistically significant.

For fungi, a random forest model explained only 19.89% of the variability of the α-diversity. The most relevant factor was the concentration of Cu, followed by the concentration of silt, Zn, total N, organic matter, and sand. A linear model showed that the concentration of Cu and Zn were negatively correlated to the α-diversity of the fungal microbiota (Additional file 8: Figure S8 and Additional file 27: Table S14). The correlations with silt, total N, organic matter, and sand were not statistically significant.

### The characteristics of the soil had a large impact on the structure of the microbiota

**Fig. 4:**
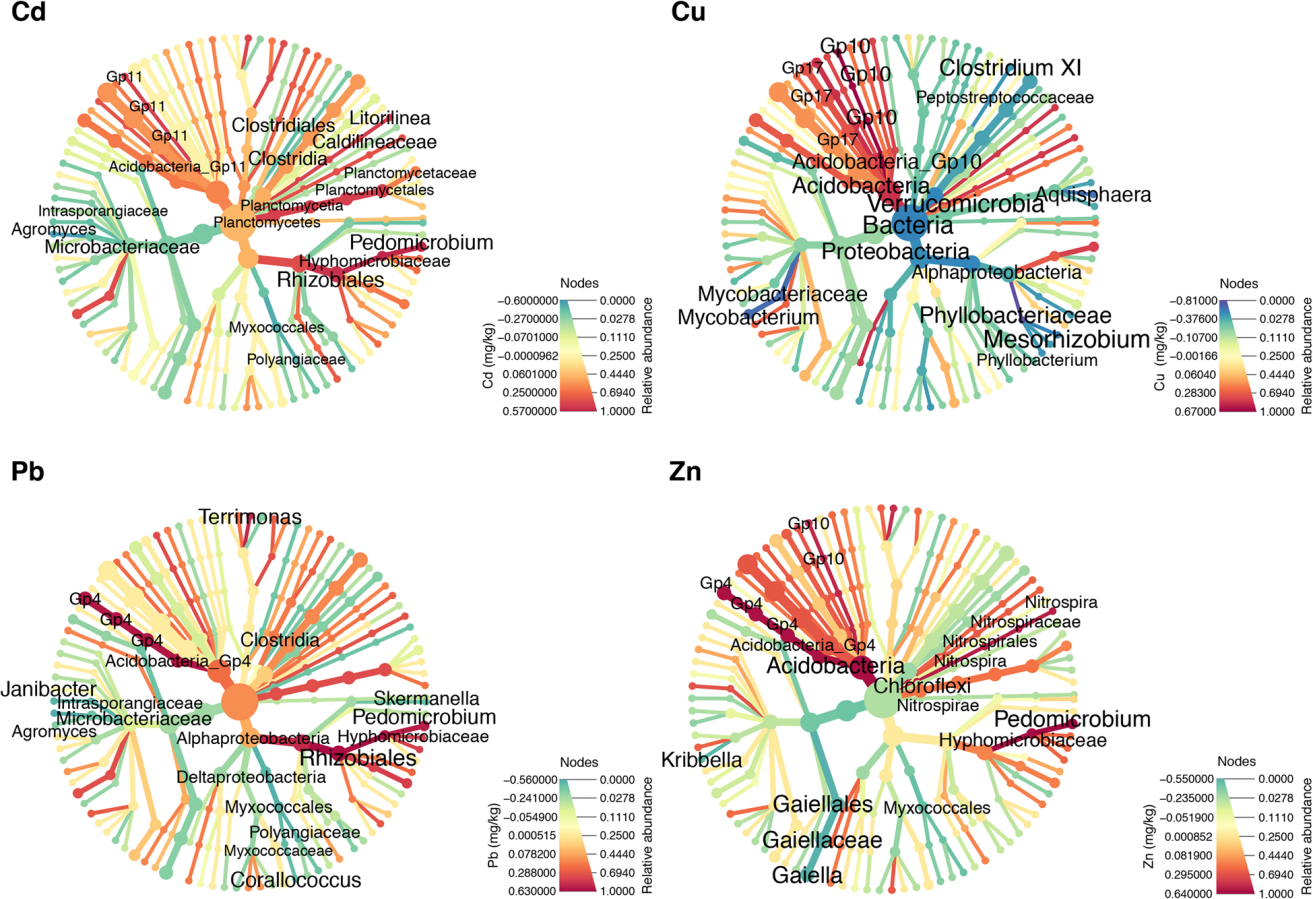
Correlation between the concentration of metals and relative abundance of bacterial taxa. The size of the nodes is proportional to the relative abundance of the taxon, while the color indicates the strength of the association measured by the Spearman rho correlation coefficient. Only taxa that were present in more than 50% of the samples are shown. The labels for the taxa with the 25 strongest association (either positive are negative) are shown. The size of the nodes is proportional to the relative abundance of each taxon, while the color indicates the strength of the association measured by the Spearman ρ. The concentration of metals has a strong influence on bacterial taxa already at high taxonomic level. In particular, the relative abundance of *Bacteria* vs *Archaea* is strongly correlated with the concentration of Cu

**Fig. 5:**
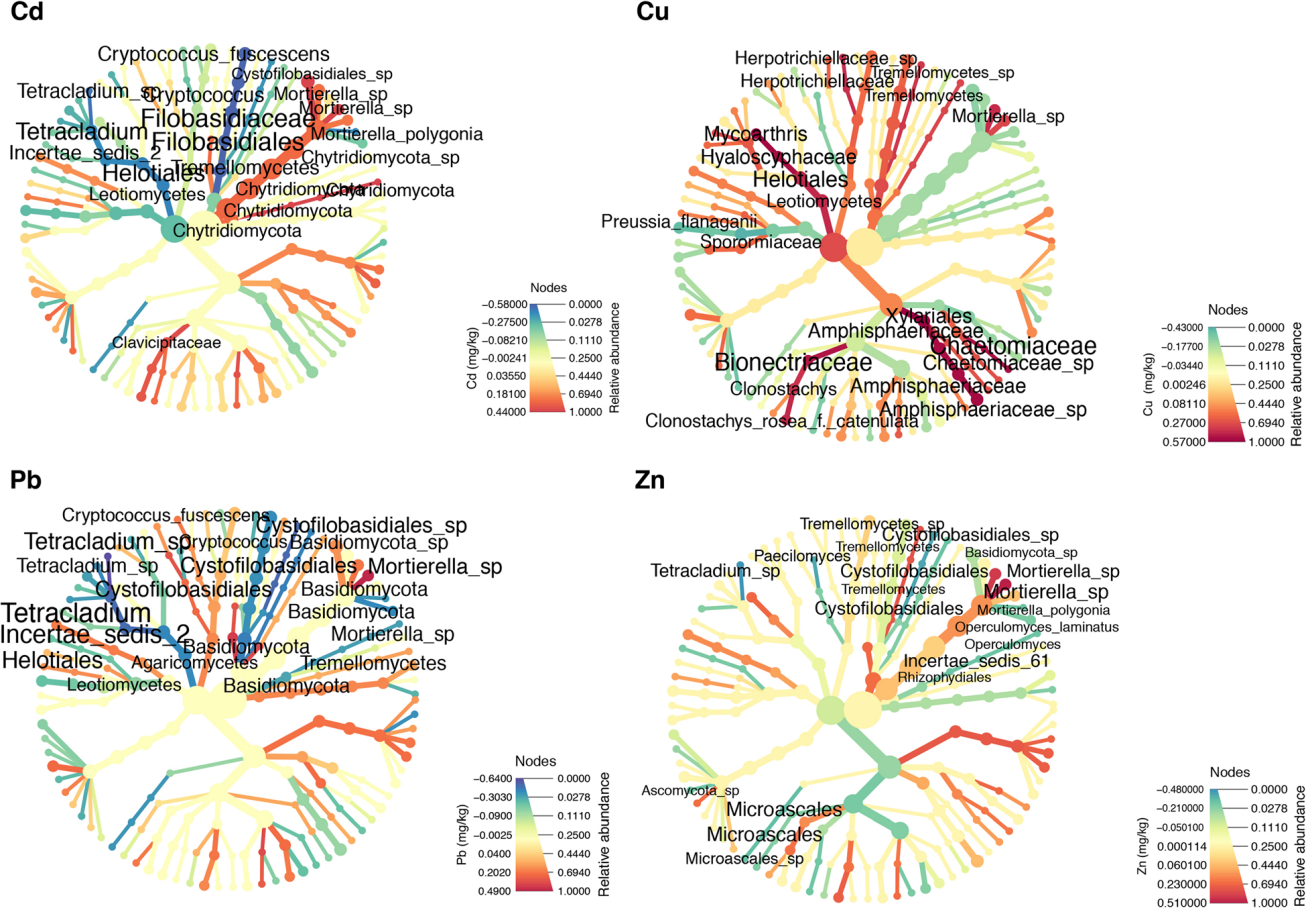
Correlation between the concentration of metals and relative abundance of fungal taxa. The size of the nodes is proportional to the relative abundance of each taxon, while the color indicates the strength of the association measured by the Spearman ρ. Only taxa that are present in more than 50% of the samples are shown

We performed a maximal information coefficient analysis [42] at all taxonomic ranks to identify taxa that were sensitive to specific characteristics of the soil (Additional file 28: Table S15 and Additional file 29: Table S16, respectively). The characteristics of the soil correlated differently with different bacterial and fungal clades (Figs. 4, 5 and Additional file 9: Figure S9 and Additional file 10: Figure S10). The ratio between the relative abundances of bacteria and archaea was associated with all the measured quantities, with the exception of the C/N ratio and the concentration of Cd. In some cases (Additional file 11: Figure S11 and Additional file 28: Table S15), these associations correspond to non-zero values of Spearman’s rho (ρ = 0.54, MICe = 0.38 for the concentration of Cu and ρ = − 0.44, MICe = 0.37 for the relative abundance of sand, respectively), while in other cases, the Spearman’s rho was close to 0 (ρ = − 0.09, MICe = 0.22 for the relative abundance of clay). In general, the strongest associations (positive and negative, respectively) in 16S data are between the relative abundances of two bacterial families, namely, the family *Verrucomicrobiaceae* of the order *Verrucomicrobiales* [46] (ρ = 0.72, MICe = 0.51) that was positively correlated with the amount of silt, and the family *Phyllobacteriaceae* [47] of the order *Rhizobiales* (ρ = − 0.81, MICe = 0.58), that was negatively correlated with the concentration of Cu (Additional file 12: Figure S12 a and b, respectively).

For fungi, several clades were strongly correlated to the characteristics of the soil (Fig. 5 and Additional file 10: Figure S10). The strongest associations were between one OTU from the family *Herpotrichiellaceae*, that was negatively correlated with the relative amount of silt (ρ = − 0.80, MICe = 0.72) and positively with the relative amount of sand (ρ = 0.71, MICe = 0.55).

### The composition of the bacterial microbiome correlates with geography, while fungal communities are dominated by cultivation type

**Fig. 6:**
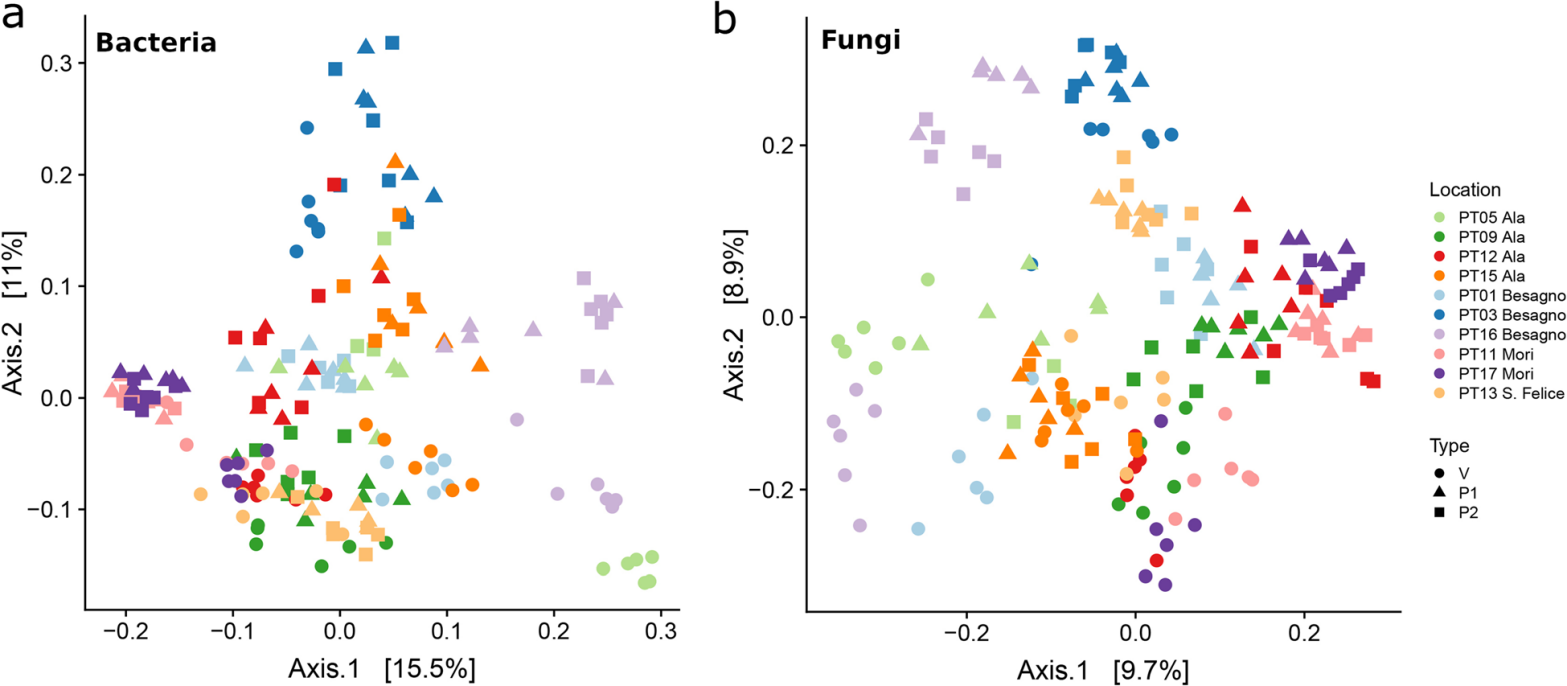
**a** PCoA of the Bray-Curtis distance matrix for bacteria. The samples separated by location, site, and type. While samples from permanent grassland soils only rarely cluster with samples from different locations, vineyard samples from different locations in some cases are closely related (e.g., PT12-Ala, PT17-Mori, and PT13-S.Felice, and PT15-Ala, PT16-Besagno). **b** PCoA and of the Bray-Curtis distance matrix for fungi. While P1 and P2 samples from the same site form well defined, closely related groups, V samples still group together, but forming clusters that are usually clearly distinct from the corresponding P1 and P2 samples. See Additional file 29: Table S16

To explore if differences in microbiome structure and composition correlate with sampling location and soil type, we computed the between-sample diversity (−diversity) using Bray-Curtis distance (Fig. 6). For bacteria, samples from the same location and type of soil generally clustered together (Fig. 6a). In addition, samples from both types of permanent grassland soil were closely related, with only one case in which a PERMANOVA test indicated a clear distinction (PT16, Additional file 29: Table S16) and two in which the test was marginally significant (*p* value = 0.01, PT05 and PT17). Differently, in all locations, the vineyard soils were clearly distinct from the corresponding permanent grassland soils (Additional file 29: Table S16). However, in most cases, samples from the same location formed well-defined groups and the distances between permanent grassland and vineyard samples from the same site were smaller than the distances between samples from different sites (Additional file 13: Figure S13).

For fungi (Fig. 6b), samples from permanent grassland soils were closely related (with two exceptions, namely, PT01 and PT16, and one, PT17, for which the PERMANOVA test was marginally significant, i.e., *p* = 0.01, Additional file 29: Table S16), and were distinct from corresponding vineyard samples. However, in contrast with what was found for bacteria, a large fraction of the samples from the vineyards formed in a large group (Fig. 6b), samples from the same site were in most cases similar to the distances between samples from different sites (Additional file 13: Figure S13). These results suggest that soil usage has a stronger effect on the fungal component of the soil microbiota than on bacteria.

## Discussion

Despite growing interest, the global and local variability of soil microbial communities and the biotic and abiotic factors that drive their differentiation are still poorly understood. Even on the local scale, soil can be composed by a large number of different habitats, that range from the immediate surroundings of plant roots (the rhizosphere) to bulk soil, where factors essential for survival of microbial communities (including, for instance, oxygen, water or nutrient availability) can vary substantially on the millimeter to centimeter scale [7, 10].

Soil microbial communities are of particular relevance in grape cultivation, since soil microbiota has been shown to serve as a reservoir of microorganisms colonizing grapes [4], contributing to shape regional wine characteristics [48].

To characterize the impact of cultivation and of other environmental factors on soil microbial communities in vineyards, we profiled via deep amplicon sequencing the bacterial and fungal components of the microbiome of soils sampled from vineyards and from associated permanent grasslands situated in close proximity in 10 sites geographically located in 4 different areas in the Adige Valley. Consistently with previous results obtained on both cultivated and permanent grassland soils [9, 49], a large fraction of the microbiome of each sample was constituted by sequences from yet uncharacterized taxa, thus supporting the notion that the soil environment is still severely underrepresented in sequence databases [50]. At the OTU level, we found a high degree of variability across samples. Most of the bacterial OTUs were present in a small number of samples, and only a small number of core OTUs were shared by all samples. However, core OTUs were the dominant fraction of all the samples in terms of relative abundance, confirming earlier works [17] that have shown that approximately 500 bacterial phylotypes accounted for nearly half of the soil communities worldwide and supporting the recent finding that the bacterial diversity found on the local scale recapitulates what is found on the global scale [49]. The size of the core of shared bacterial OTUs increased significantly when we considered more homogenous samples, both in terms of geographical location or land use, showing that both dispersion limitations and agricultural practices influence the bacterial component of the bulk soil microbiota.

Surprisingly, we found that the variability of fungal communities followed patterns that were qualitatively different from what was found for bacteria. Using the ITS data from the same samples, we estimated that the core mycobiome was limited to less than 5 OTUs, accounting on average for approximately 15% of the sequences of each sample. Despite the relatively limited geographical range sampled in this study, these results are in striking agreement with the dominant role of a small number of taxa, in particular from the phylum *Ascomycota*, that has been highlighted by a global survey of soil samples [51]. In addition, we found that geographical location, but not land use, had an impact on determining the size of the core soil mycobiome, indicating the importance of spatial processes in structuring the biogeographic pattern of soil fungal communities [52] in accordance with what found in grape associated bacterial and fungal communities [53], and confirming that dispersal limitations play a crucial role in determining the diversity across fungal communities even on a local geographical scale [8].

This difference between bacteria and fungi, the two dominant components of the soil microbiome [7], likely reflects the general characteristics of the different dispersal behaviors of these two classes of organisms. It has been previously suggested that the distribution of fungi exhibit strong biogeographic patterns that could be driven by dispersal limitations [13, 54], while bacteria are believed to have weak biogeographical patterns. Our results support the idea that while a core set of phylotypes that dominate soil bacterial microbiota from the local to the global scale [17], for fungi the set of core phylotypes is much more limited both in terms of number and of relative abundance, and is dominated by generalist taxa that can be disseminated by wind [51]. Comparison of the microbial diversity between vineyards and associated permanent grasslands suggests that the impact of cultivation on soil microbiota strongly depends on specific characteristics of the soil and possibly of the land management. The absence of a deterministic trend induced by different uses of similar soils is consistent with earlier studies that have found no general impact on the bacterial richness of long-term land-use change [15] and of cropping system [55]. However, in other cases, an increase in soil bacterial richness was found after the conversion of forest to agriculture [56], suggesting that the effect of cultivation strongly depends on the nature of the soil and cultivation type. For fungi, agricultural intensification has been shown to be the cause of reduced connectivity of interaction networks [57]. Despite the caution that should be used when interpreting co-occurrence networks inferred from microbiome data [58], this is an indication that farming destabilizing fungal soil communities, selecting some species over others [59] and possibly leading to a general instability of the community [60].

Interestingly, we identified a widespread (8 out of 10 sampling locations) inverse correlation between the diversity of the bacterial and fungal components of the soil microbiota. The correlations between fungal and bacterial richness in environmental samples have not been studied in detail, and likely depend both on the specific characteristics of the environment under study and on general mechanisms of interactions between these two groups of organisms. The number of fungal and bacterial OTUs was positively correlated in a study of the dust-associated microbiome on the continental scale [61]. Other studies have shown that bacterial and fungal diversity correlate in an opposite way to latitude on the global scale [12], and with other environmental variables in soils in an alpine grassland ecosystem [62]. Recently, the correlation between antibiotic-resistance genes and the ratio between bacteria and fungi suggested a strong competition between the bacterial and fungal components of the microbiota through the production of antimicrobial substances from the latter [12]. The negative correlation between the fungal and bacterial diversity found here is consistent with a scenario where a diverse fungal community produces a range of antimicrobial molecules that in turn pose a strong selective pressure on bacteria [22]. Although definitive conclusions can be obtained only by correlating absolute abundances, and the fact that inferred microbial interactions can be biased by sample heterogeneity [63], our results highlight the role of fungal-bacterial interactions in determining the structure of the soil microbiota, suggesting that studies that aim at identifying the environmental factors that influence one of the two components should also take the other into account. The chemical and textural characteristics of soil partially determine the richness of the soil microbiota, and the link between the characteristics of the soil and the richness of the microbial communities is weaker for fungi than for bacteria. These results are consistent with earlier studies that have shown that the assembly of microbial communities is only partially determined by environmental conditions [54]. Individual taxa are influenced differently by physico-chemical characteristics of the soil, supporting the hypothesis that the microbiome structure can be manipulated [16].

Considering the between-samples variability, we found that the effect of land use on the structure and composition of the microbiome was especially strong for the fungal component, while for bacteria the geographical origin of the samples was the dominant factor. This result is in apparent contrast with the result that geography was the only relevant factor determining the size of the core of fungal species conserved across samples. However, the fungal component of the microbiota in samples from the vineyard was characterized by the presence of one OTU representing a large fraction of the sequences, distinguishing them from other samples without altering significantly the core size in terms of shared OTUs. This OTU was classified as a member of the *Amphisphaeriaceae*, a family of Ascomycota that includes both plant pathogens and endophytes [64, 65] and that has been shown to colonize grapevine wood [66] and survive to sterilization through hot water treatment [67]. This association with grapevine is probably the cause of the relatively high abundance of this fungus in vineyard samples, suggesting that the identity of the cultivated species, more than cultivation itself, has a strong influence on the fungal component of the soil microbiome.

## Supplementary information

**Additional file 1: Figure S1.** Schematic representation of sampling area. Each field was divided in 3 portions: the zone with grapevines (V) and two permanent grasslands, P1 and P2_,_ respectively at 8 and 16 m from V; 6 replicates were made for each portion (black crosses). P1 and P2 represent portions of fields with a minimum human intervention, without tractors passage or chemical treatments, only mowing (twice per year). (JPG 231 kb)

**Additional file 2: Figure S2.** Geographical maps of sampling sites. The left panel reports all sampling sites compared to the whole Trentino-Alto Adige region, positions are then detailed in the right panel. The altitude respect to the sea level is represented as grayscale. “PT” prefix for sampling sites is omitted for sake of clarity. (JPG 735 kb)

**Additional file 3: Figure S3.** PCA of the chemical characteristics of the samples. (JPG 640 kb)

**Additional file 4: Figure S4.** a) Log_2_ Fold Change of bacterial OTUs with relative abundances significantly different between vineyards and associated uncultivated fields. b) Relative abundances of the top ten bacterial OTUs whose relative abundances change most significantly between vineyards and associated permanent grassland. Only Otus with more than 10 reads in 25% of the samples are shown. (JPG 2364 kb)

**Additional file 5: Figure S5.** a) Log2 Fold Change of fungal OTUs with relative abundances significantly different between vineyards and associated permanent grassland. b) Relative abundances of the top ten fungal OTUs whose relative abundances change most significantly between vineyards and associated permanent grassland. Only Otus with more than 10 reads in 25% of the samples are shown. (JPG 2076 kb)

**Additional file 6: Figure S6.** Size of the core bacterial a) amd fungal b) microbiome as a function of the number of samples. The red line is a power law fit. (JPG 360 kb)

**Additional file 7: Figure S7.** α-diversity of the bacterial microbiota as a function of the six most relevant features identified by random forest: CaCo_3_ (A), Sand (B), Silt (C), Zinc (D), Copper (E) and pH (F). The blue line is a maximum likelihood linear fit, the shaded area is the 95% confidence interval of the linear model. Parameters of the fit are reported in Additional file 26: Table S13. (JPG 934 kb)

**Additional file 8: Figure S8.** α-diversity of the fungal microbiota as a function of the six most relevant features identified by random forest: Copper (A), Silt (B), Zinc (C), Nitrogen (D), Organic matter (E), sand (F). The blue line is a maximum likelihood linear fit, the shaded area is the 95% confidence interval of the linear model. Parameters of the fit are reported in Additional file 27: Table S14. (JPG 922 kb)

**Additional file 9: Figure S9.** Correlation (Spearman rho) between the soil texture and chemical characteristics and the relative abundance of bacterial taxa. The size of the nodes is proportional to the relative abundance of the taxon, while the color indicates the strength of the association measured by the Spearman Rho correlation coefficient. Only taxa that are present in more than 50% of the samples are shown. (JPG 3518 kb)

**Additional file 10: Figure S10.** Correlation (Spearman rho) between the soil texture and chemical characteristics and the relative abundance of fungal taxa. The size of the nodes is proportional to the relative abundance of the taxon, while the color indicates the strength of the association measured by the Spearman Rho correlation coefficient. Only taxa that are present in more than 50% of the samples are shown. (JPG 3071 kb)

**Additional file 11: Figure S11.** Relative abundance of OTUs from the Kingdom *Bacteria* vs soil characteristics. Lines are *loess* smoothing of the data, shaded areas are the 95% confidence intervals around the smooth. (JPG 2712 kb)

**Additional file 12: Figure S12.** a) Relative abundance of the Family *Phyllobacteriaceae* as a function of the concentration of Cu. b) Relative abundance of the Family *Verrucomicrobiaceae* as a function of the concentration of Silt. The line is a *loess* smoothing of the data, and the shaded area is the 95% confidence interval. (JPG 726 kb)

**Additional file 13: Figure S13.** a) Boxplot of the Bray Curtis distances between the bacterial component of the microbiota of samples within the permanent grassland (P) and vineyard (V) soils and between vineyard and grassland soils (V vs P) for each site. b) same as a, across different sites. c) Same as a, for fungi. d) Same as b, for fungi. (JPG 47 kb)

**Additional file 14: Table S1.** Main features of sample fields (A and B). All samples were from vineyards of Adige valley in Trentino region and sampled the very same day (July the 12th of 2017). Legend. V = vineyard, P1 = grassland at 8 m from V, P2 = grassland at 16 m from V (see also Additional file 1: Figure S1). Loc. Name = Location Name, Trellis sys. = Trellis system, m asl = meters at sea level. (DOCX 15 kb)

**Additional file 15: Table S2.** Results of the Deseq analysis of bacterial taxa significantly differentially abundant in vineyard vs permanent grassland (P_1_ and P_2_ are considered together) samples for 16S data. (XLSX 53 kb)

**Additional file 16: Table S3.** Results of the Deseq analysis of fungal taxa significantly differentially abundant in vineyard vs permanent grassland (P_1_ and P_2_ are considered together) samples for ITS data. (XLSX 22 kb)

**Additional file 17: Table S4.** The site-specific core microbiome for Bacteria and Archaea, and Fungi. (DOCX 14 kb)

**Additional file 18: Table S5.** Pairwise comparison of location α-diversity measured by the Shannon Entropy for bacteria A) and fungi B) using the Wilcoxon rank-sum test, FDR corrected. Highlighted in bold values below the significance threshold of 0.05. (DOCX 14 kb)

**Additional file 19: Table S6.** Pairwise comparison of site α-diversity measured by the Shannon Entropy for bacteria a) and fungi b) using the Wilcoxon rank-sum test, FDR corrected. Comparisons are ordered by sampling area (PT13 is the only sample from S. Felice area). Highlighted in bold values below the significance threshold of 0.05 while light gray shade enlight comparisons inside the same area. (DOCX 17 kb)

**Additional file 20: Table S7.** Average differences of the α-diversity of bacterial and fungal communities measured by the Shannon entropy between the Vineyards and P1 samples for each site. Statistically significant contrasts are highlighted in bold. While for bacteria in no case the P1 and P2 samples have significantly different richness, for fungi, in two cases the α-diversity of the P2 samples are significantly different from the P1 samples (higher in PT16 and lower in PT17, *p*-values 0.00116 and 0.040, respectively). (DOCX 14 kb)

**Additional file 21: Table S8.** Linear model correlating the the bacterial and fungal α-diversities in all sites. (DOCX 14 kb)

**Additional file 22: Table S9.** Linear model correlating the the bacterial and fungal α-diversities, stratified by site. (DOCX 14 kb)

**Additional file 23: Table S10.** Linear model correlating the the bacterial and fungal α-diversities for PT05. (DOCX 13 kb)

**Additional file 24: Table S11.** Linear model correlating the the bacterial and fungal α-diversities for PT12. (DOCX 13 kb)

**Additional file 25: Table S12.** Linear mixed effect model correlating the the bacterial and fungal α-diversities for all sites except PT05 and PT12. Intercept is a treated as a random effect, slope as a fixed effect. (DOCX 13 kb)

**Additional file 26: Table S13.** Parameters of the linear models in Additional file 6: Figure S6 modeling the richness of bacterial microbiota (Shannon entropy) against the chemical characteristics of the soil. (DOCX 14 kb)

**Additional file 27: Table S14.** Parameters of the linear models in Additional file 7: Figure S7 modeling the richness of fungal microbiota (Shannon entropy) against the chemical characteristics of the soil. (DOCX 14 kb)

**Additional file 28: Table S15.** Taxa significantly correlated with chemical and physical characteristics of the soil from 16S data and ITS data. (XLSX 654 kb)

**Additional file 29: Table S16.** PERMANOVA results for the distinction between P1 and P2 (left) and P1 + P2 and V (right) for bacteria and fungi. In the P1 + P2 vs V comparison the P1 and P2 samples were considered together. Significant p-values (*p* < 0.01) are marked in bold. (DOCX 14 kb)

## Data Availability

Raw sequencing data are available at the European Nucleotide Archive (https://www.ebi.ac.uk/ena) under the study id PRJEB31356.
