# Supplementary figures and images for: Microbiome of vineyard soils is shaped by geography and management
Source: Microbiome. 2019 Nov 8;7:140. doi: 10.1186/s40168-019-0758-7 (PMC6839268; doi:10.1186/s40168-019-0758-7)

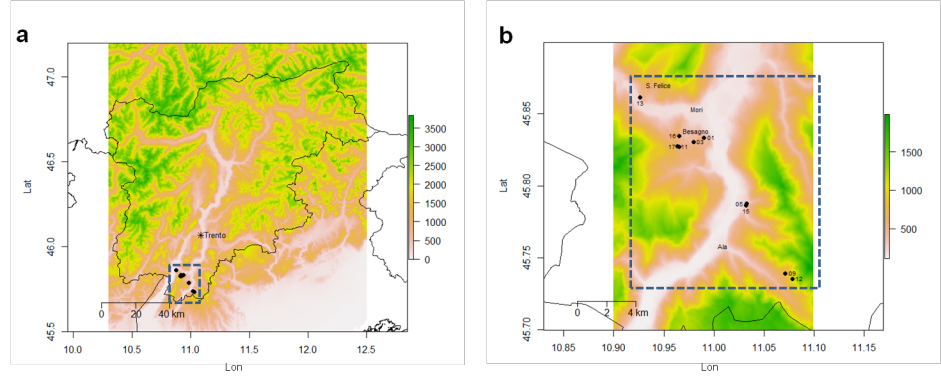

Supplement: Supplementary file 1 — Additional file 1: Figure S1. Schematic representation of sampling area. Each field was divided in 3 portions: the zone with grapevines (V) and two permanent grasslands, P1 and P2, respectively at 8 and 16 m from V; 6 replicates were made for each portion (black crosses). P1 and P2 represent portions of fields with a minimum human intervention, without tractors passage or chemical treatments, only mowing (twice per year). (JPG 231 kb) [file 40168_2019_758_MOESM1_ESM.jpg]

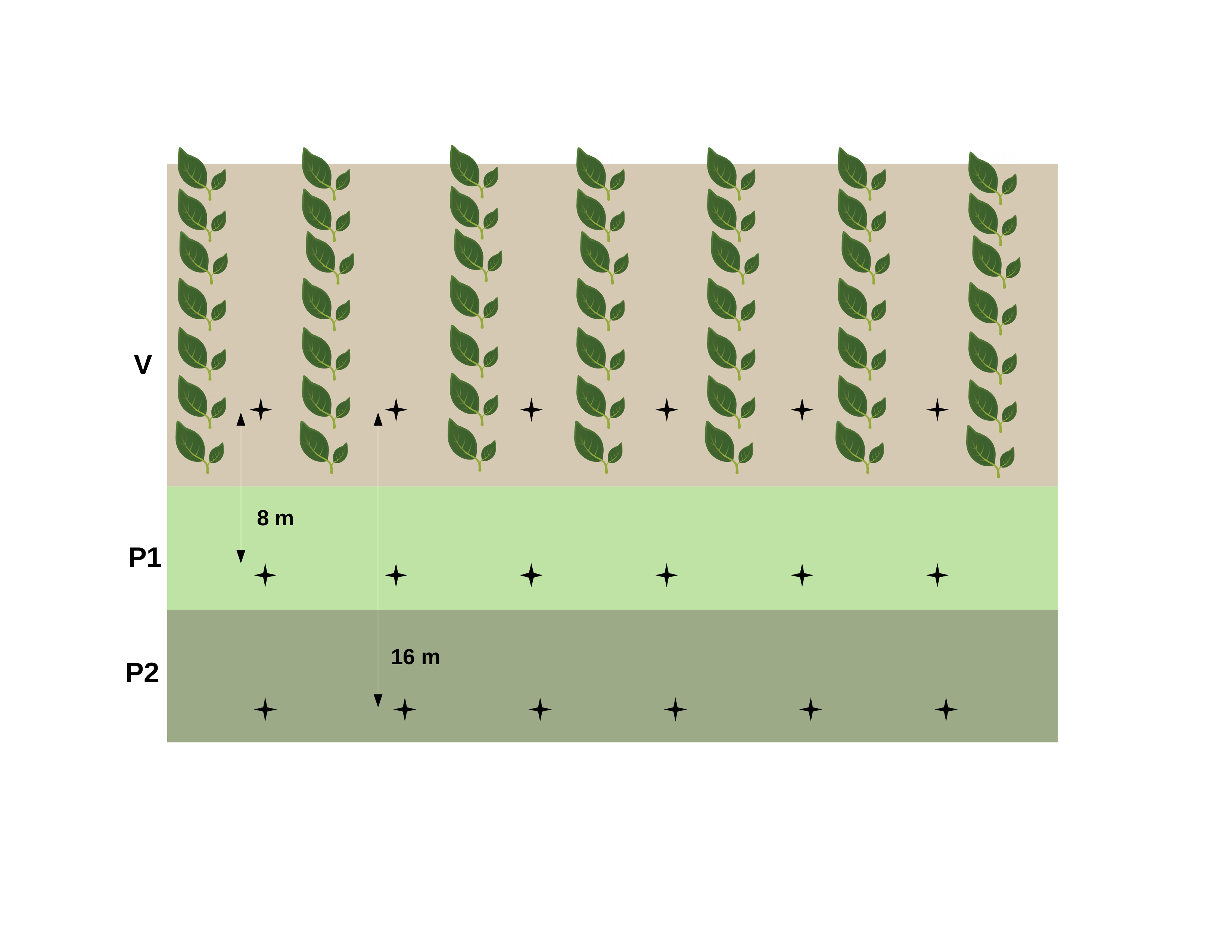

Supplement: Supplementary file 2 — Additional file 2: Figure S2. Geographical maps of sampling sites. The left panel reports all sampling sites compared to the whole Trentino-Alto Adige region, positions are then detailed in the right panel. The altitude respect to the sea level is represented as grayscale. “PT” prefix for sampling sites is omitted for sake of clarity. (JPG 735 kb) [file 40168_2019_758_MOESM2_ESM.jpg]

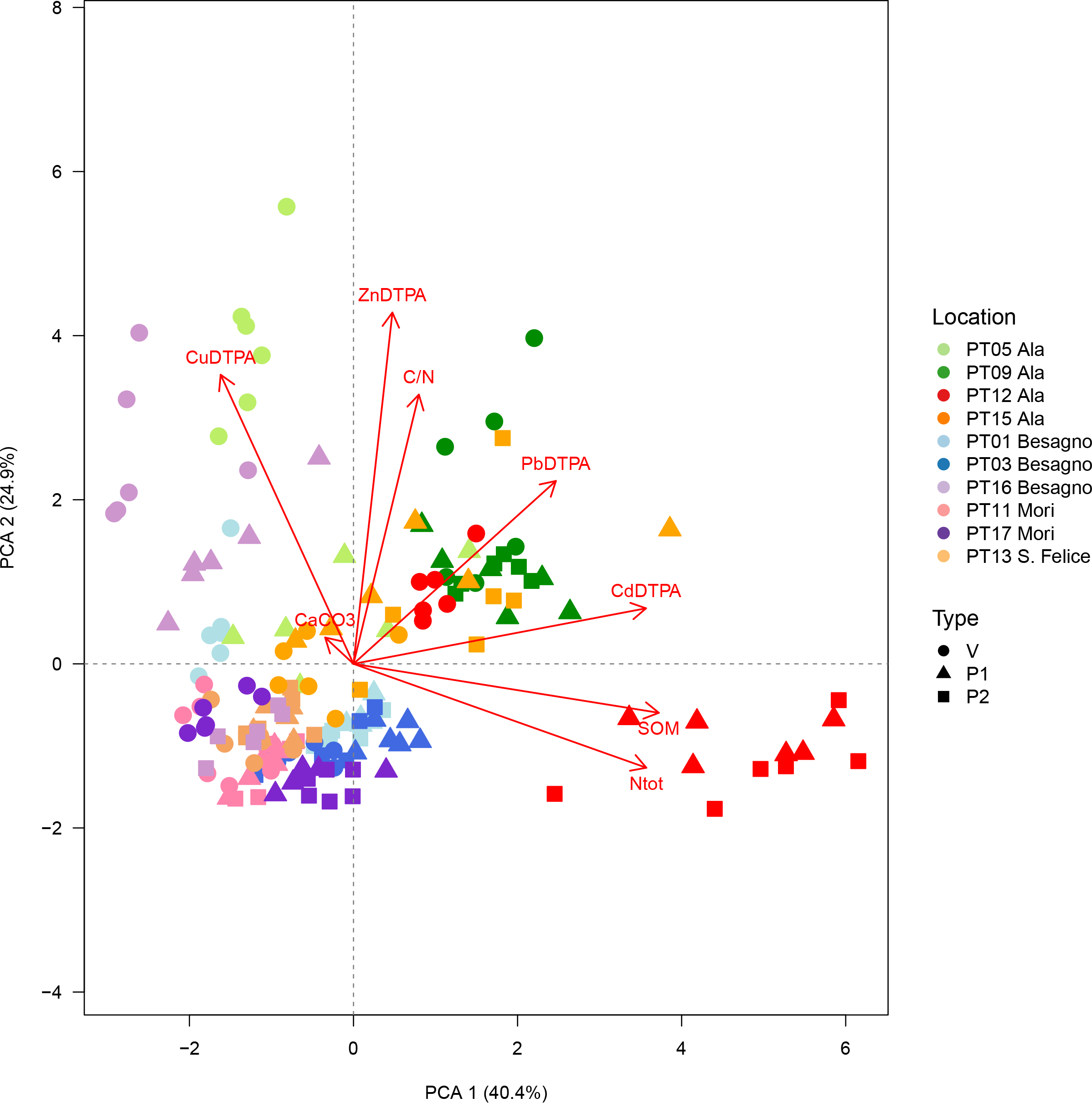

Supplement: Supplementary file 3 — Additional file 3: Figure S3. PCA of the chemical characteristics of the samples. (JPG 640 kb) [file 40168_2019_758_MOESM3_ESM.jpg]

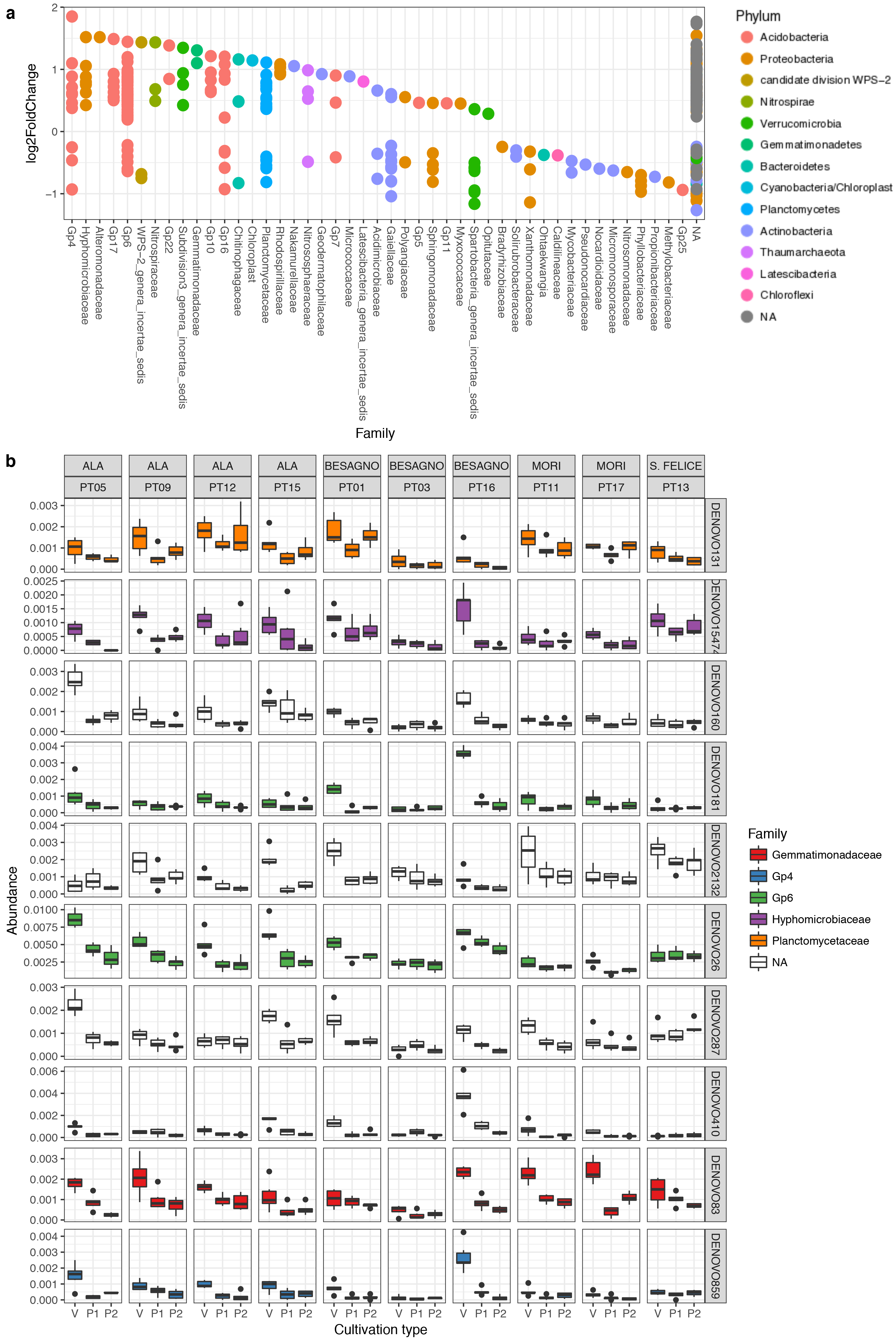

Supplement: Supplementary file 4 — Additional file 4: Figure S4. a) Log2 Fold Change of bacterial OTUs with relative abundances significantly different between vineyards and associated uncultivated fields. b) Relative abundances of the top ten bacterial OTUs whose relative abundances change most significantly between vineyards and associated permanent grassland. Only Otus with more than 10 reads in 25% of the samples are shown. (JPG 2364 kb) [file 40168_2019_758_MOESM4_ESM.jpg]

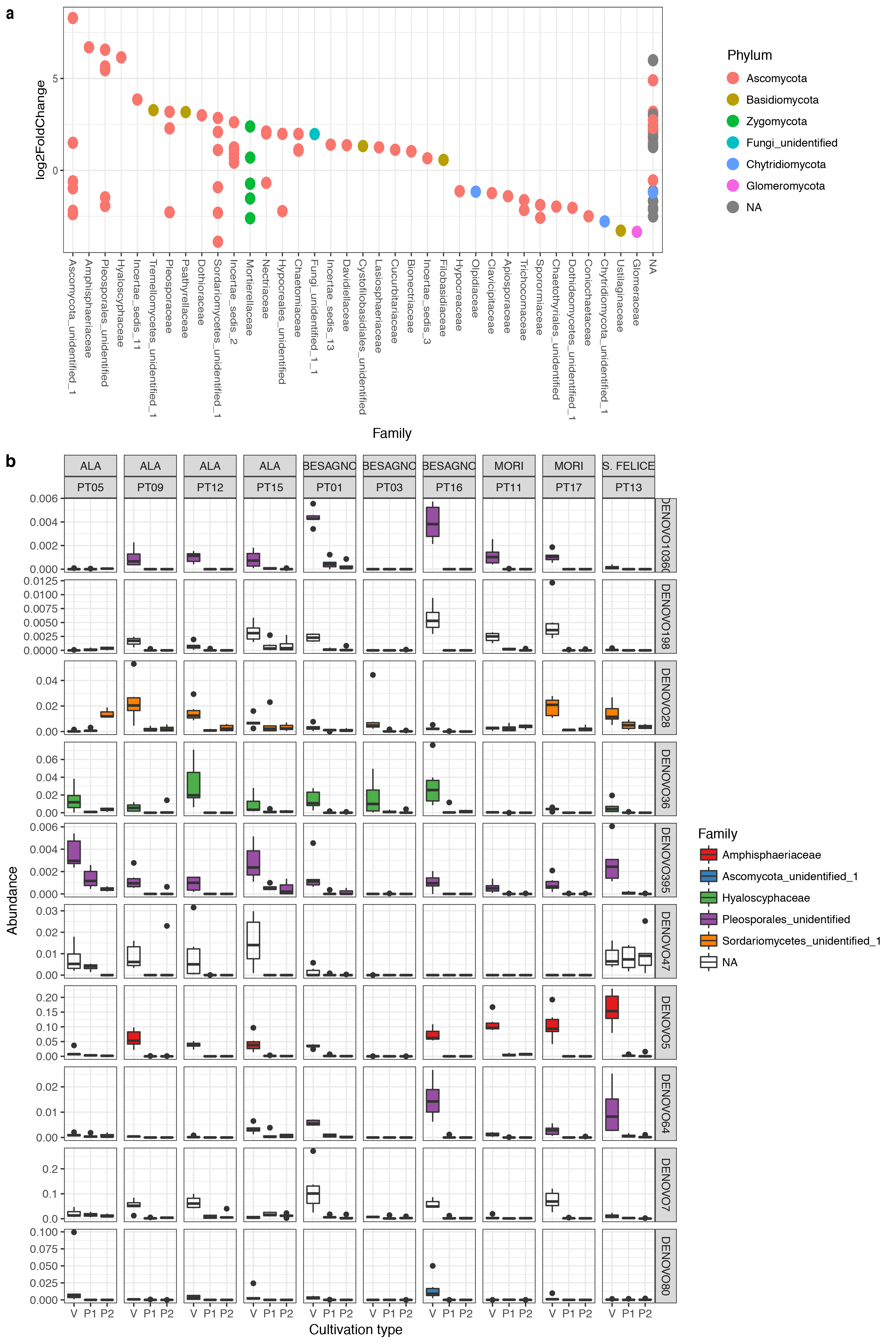

Supplement: Supplementary file 5 — Additional file 5: Figure S5. a) Log2 Fold Change of fungal OTUs with relative abundances significantly different between vineyards and associated permanent grassland. b) Relative abundances of the top ten fungal OTUs whose relative abundances change most significantly between vineyards and associated permanent grassland. Only Otus with more than 10 reads in 25% of the samples are shown. (JPG 2076 kb) [file 40168_2019_758_MOESM5_ESM.jpg]

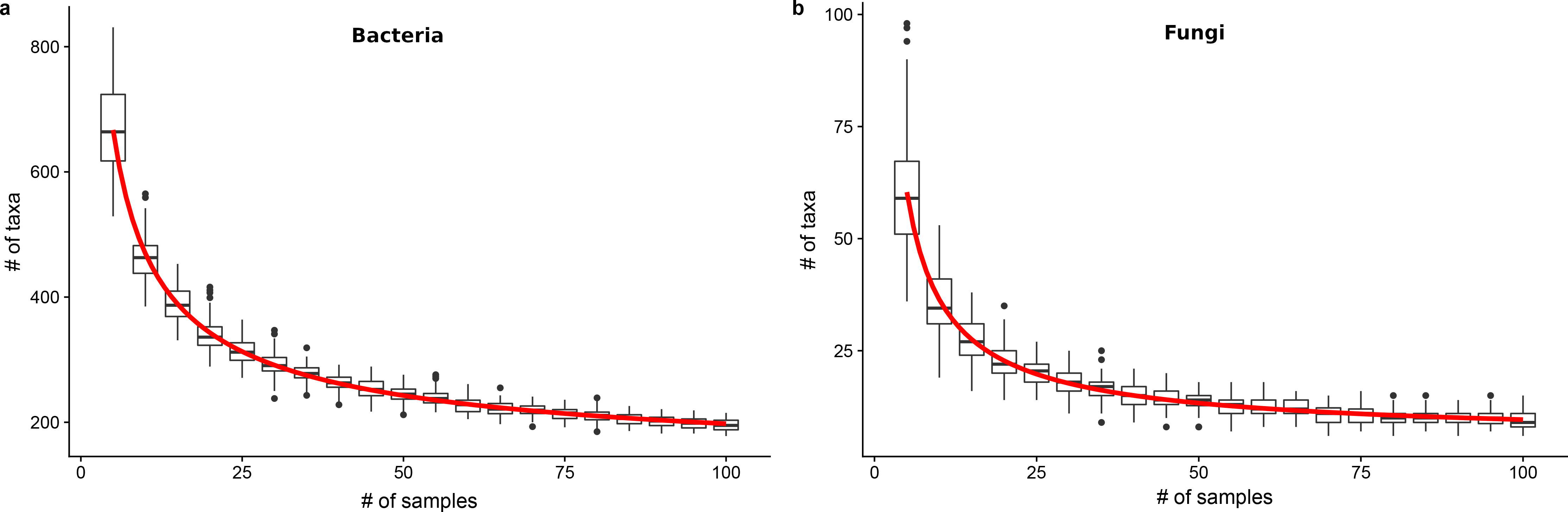

Supplement: Supplementary file 6 — Additional file 6: Figure S6. Size of the core bacterial a) amd fungal b) microbiome as a function of the number of samples. The red line is a power law fit. (JPG 360 kb) [file 40168_2019_758_MOESM6_ESM.jpg]

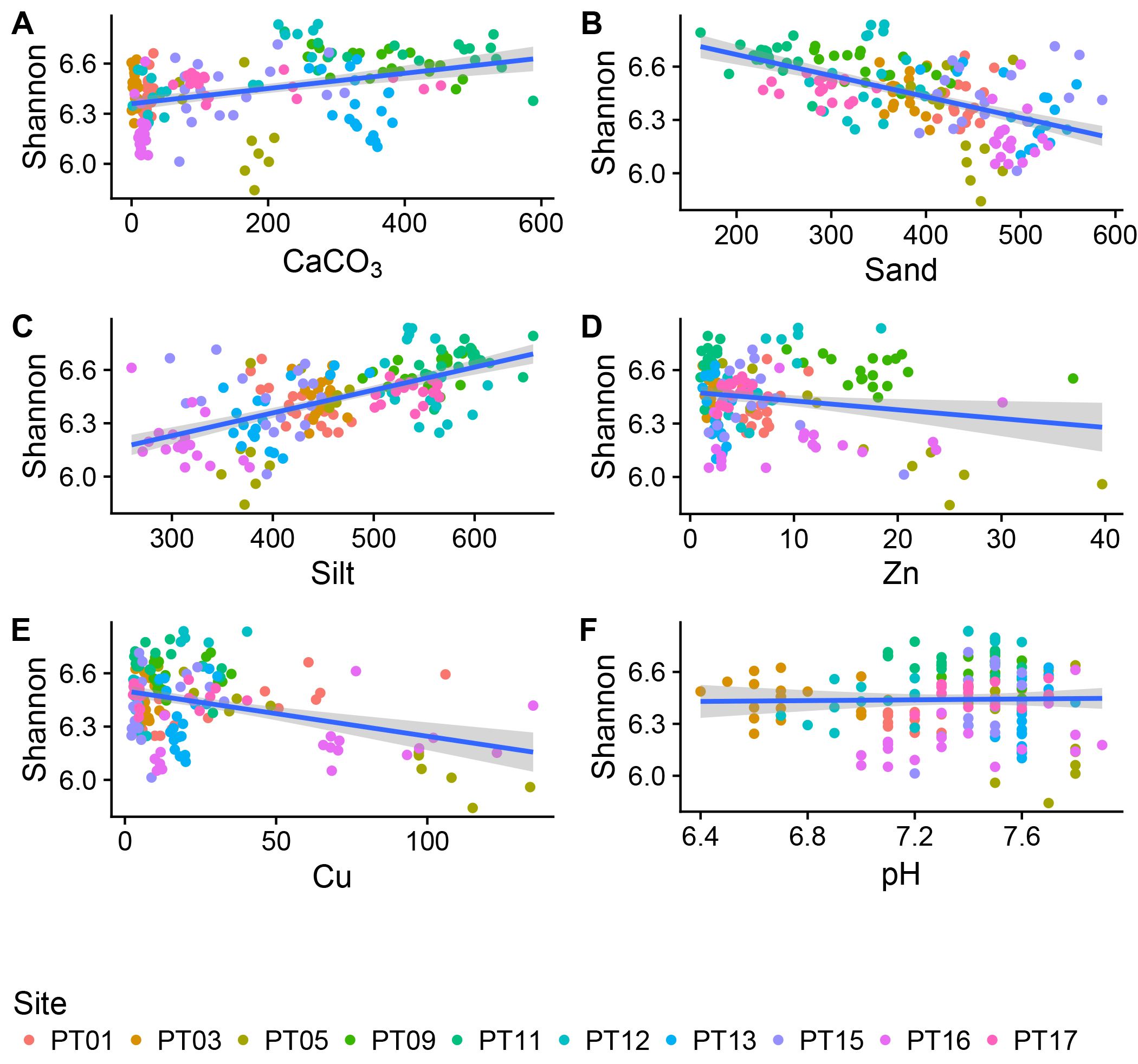

Supplement: Supplementary file 7 — Additional file 7: Figure S7. α-diversity of the bacterial microbiota as a function of the six most relevant features identified by random forest: CaCo3 (A), Sand (B), Silt (C), Zinc (D), Copper (E) and pH (F). The blue line is a maximum likelihood linear fit, the shaded area is the 95% confidence interval of the linear model. Parameters of the fit are reported in Additional file 26: Table S13. (JPG 934 kb) [file 40168_2019_758_MOESM7_ESM.jpg]

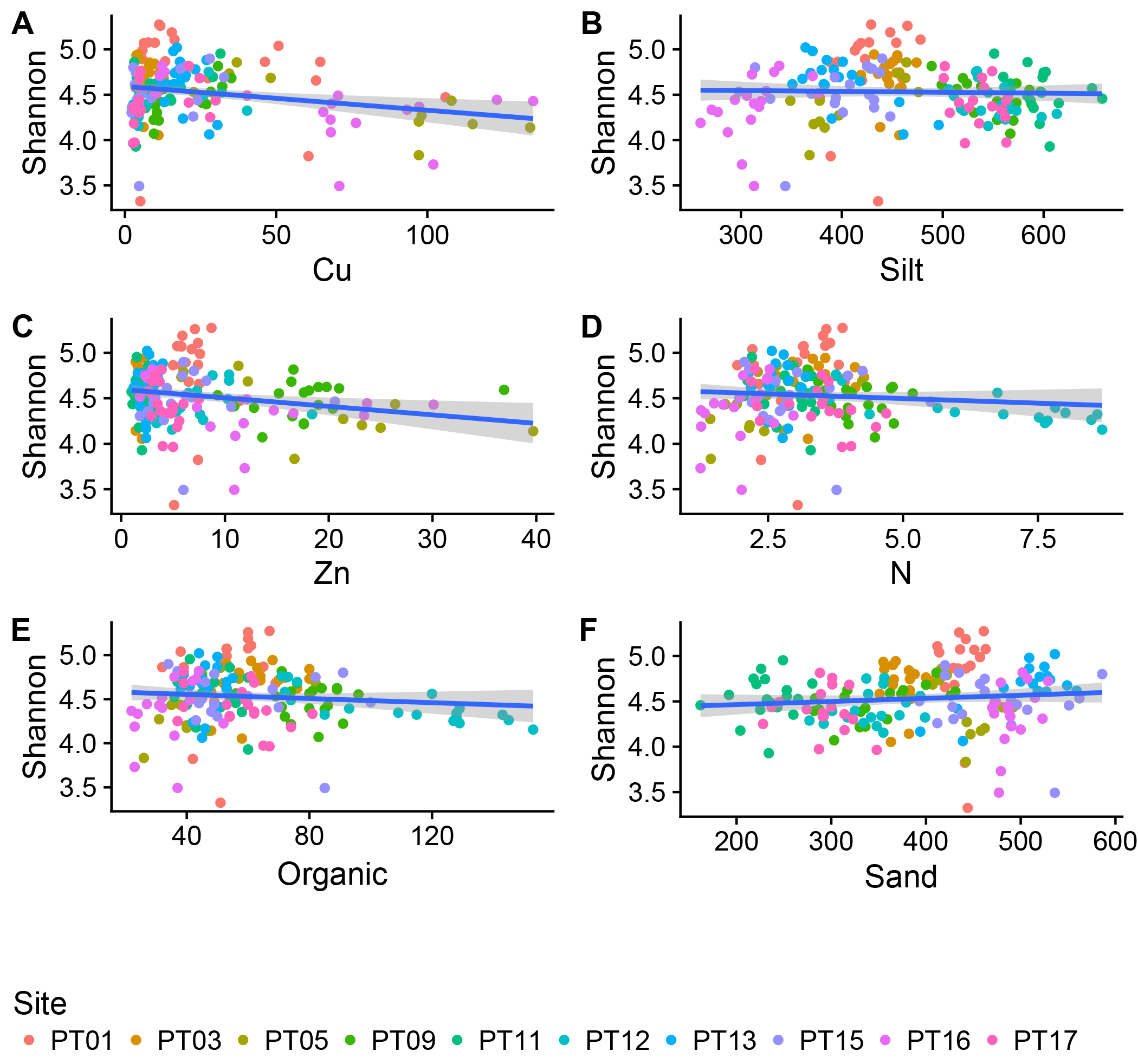

Supplement: Supplementary file 8 — Additional file 8: Figure S8. α-diversity of the fungal microbiota as a function of the six most relevant features identified by random forest: Copper (A), Silt (B), Zinc (C), Nitrogen (D), Organic matter (E), sand (F). The blue line is a maximum likelihood linear fit, the shaded area is the 95% confidence interval of the linear model. Parameters of the fit are reported in Additional file 27: Table S14. (JPG 922 kb) [file 40168_2019_758_MOESM8_ESM.jpg]

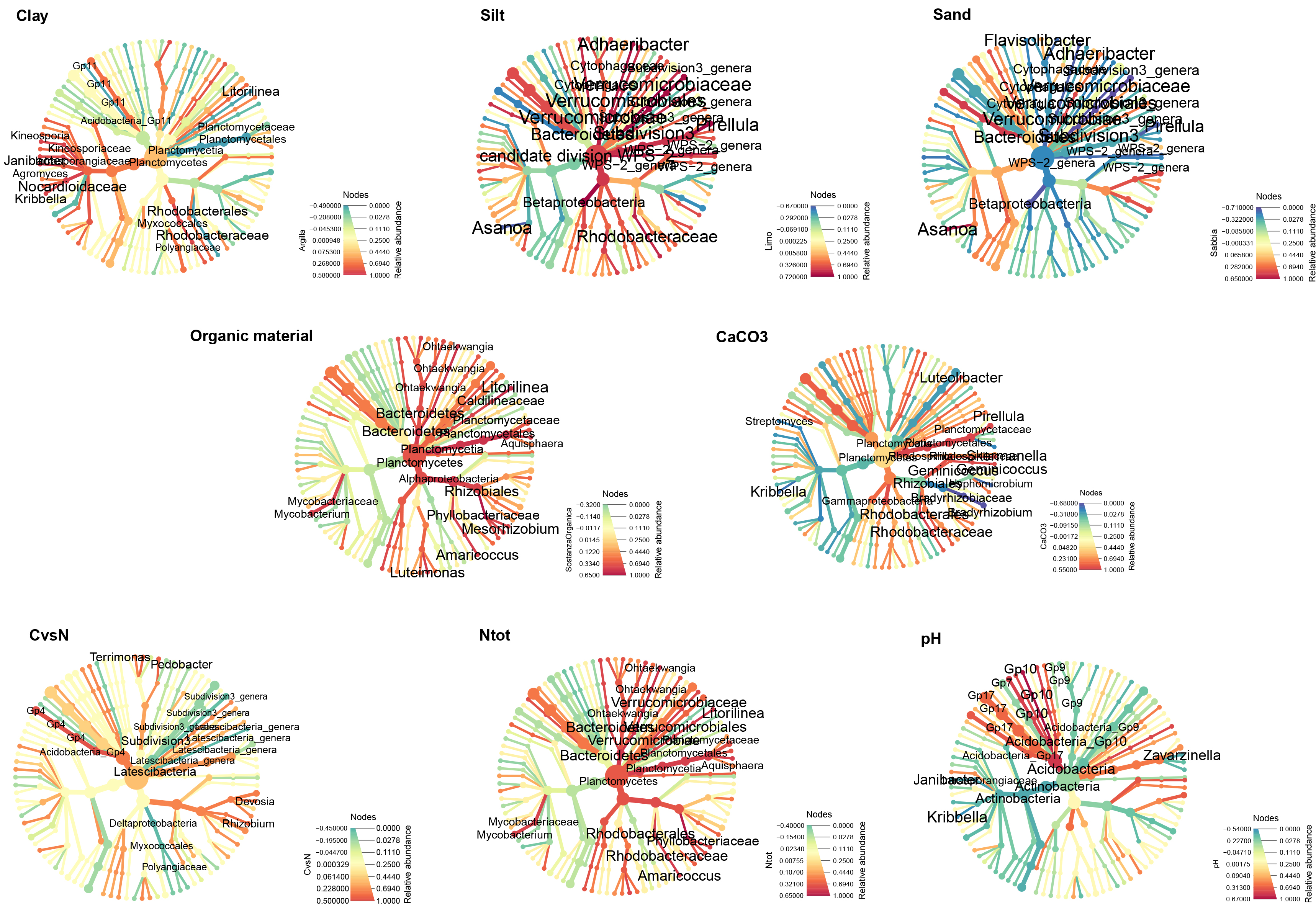

Supplement: Supplementary file 9 — Additional file 9: Figure S9. Correlation (Spearman rho) between the soil texture and chemical characteristics and the relative abundance of bacterial taxa. The size of the nodes is proportional to the relative abundance of the taxon, while the color indicates the strength of the association measured by the Spearman Rho correlation coefficient. Only taxa that are present in more than 50% of the samples are shown. (JPG 3518 kb) [file 40168_2019_758_MOESM9_ESM.jpg]

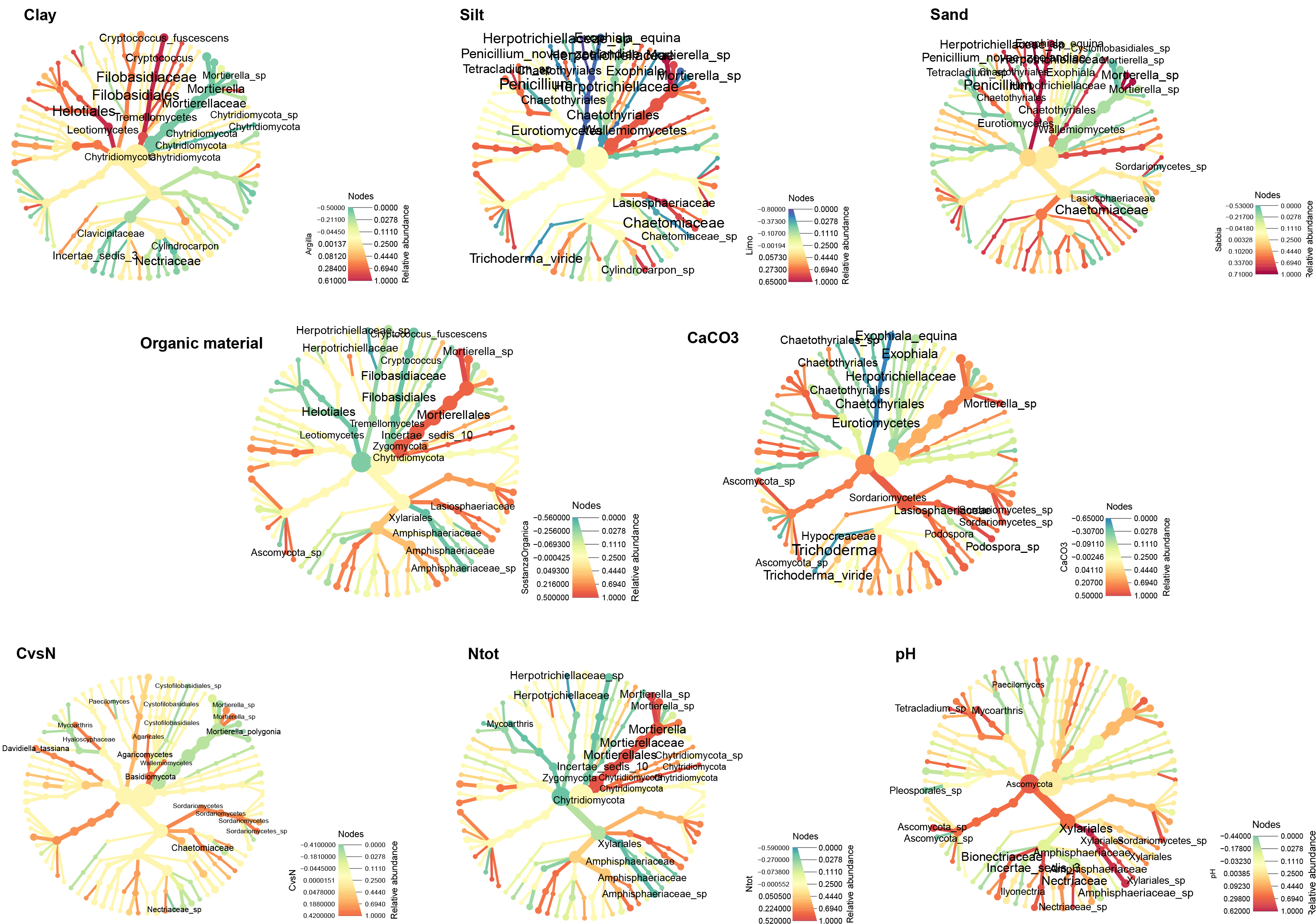

Supplement: Supplementary file 10 — Additional file 10: Figure S10. Correlation (Spearman rho) between the soil texture and chemical characteristics and the relative abundance of fungal taxa. The size of the nodes is proportional to the relative abundance of the taxon, while the color indicates the strength of the association measured by the Spearman Rho correlation coefficient. Only taxa that are present in more than 50% of the samples are shown. (JPG 3071 kb) [file 40168_2019_758_MOESM10_ESM.jpg]

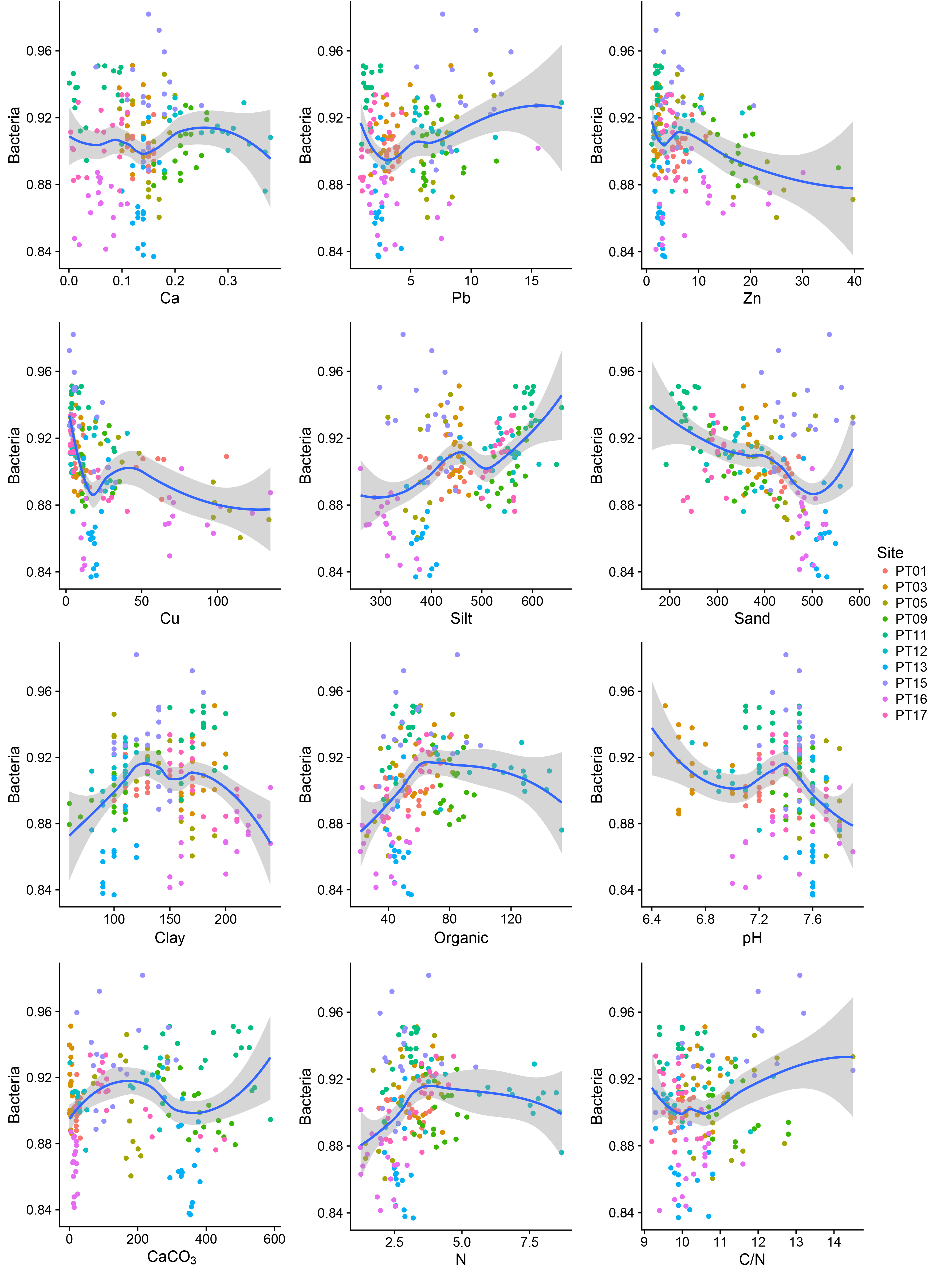

Supplement: Supplementary file 11 — Additional file 11: Figure S11. Relative abundance of OTUs from the Kingdom Bacteria vs soil characteristics. Lines are loess smoothing of the data, shaded areas are the 95% confidence intervals around the smooth. (JPG 2712 kb) [file 40168_2019_758_MOESM11_ESM.jpg]

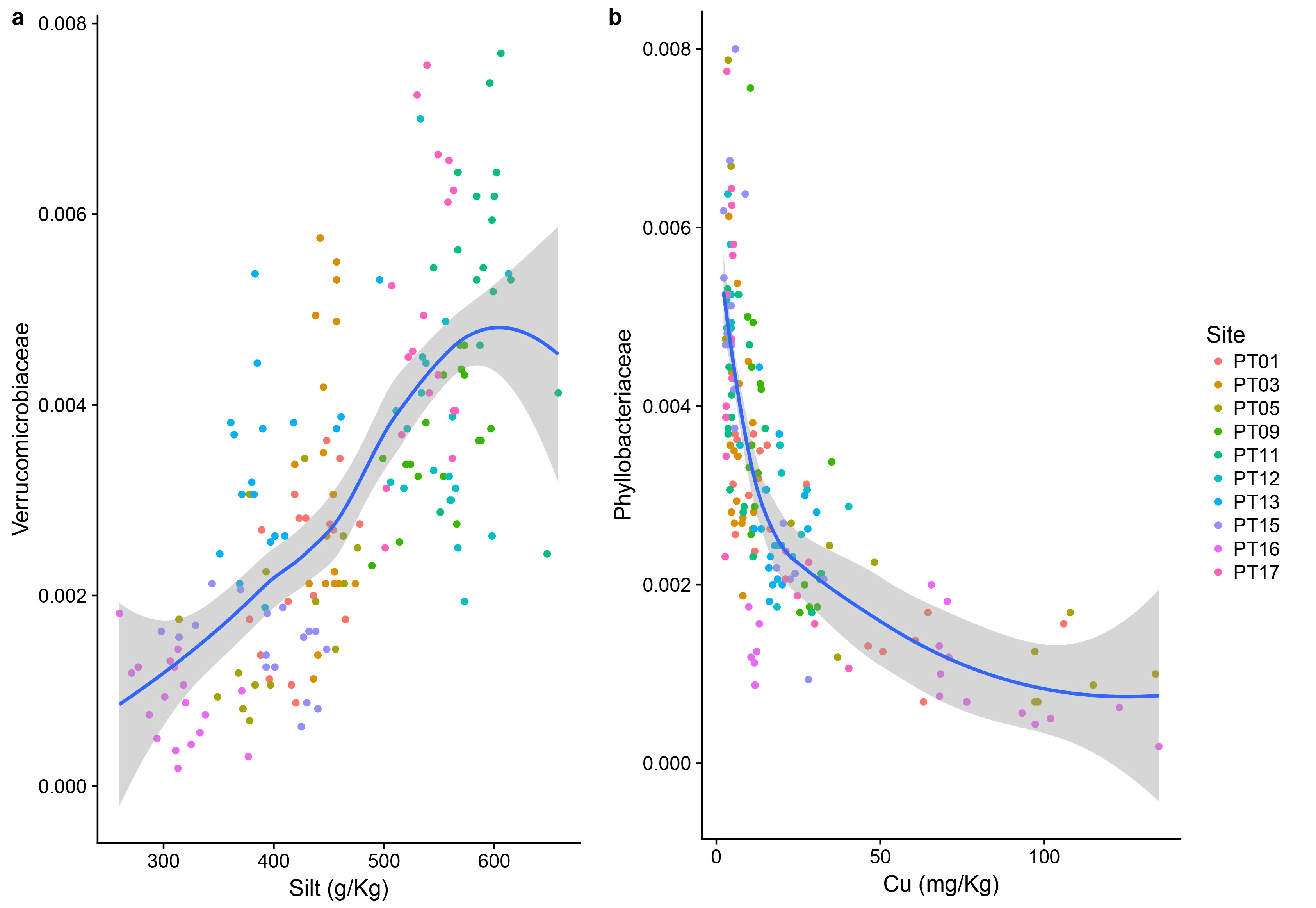

Supplement: Supplementary file 12 — Additional file 12: Figure S12. a) Relative abundance of the Family Phyllobacteriaceae as a function of the concentration of Cu. b) Relative abundance of the Family Verrucomicrobiaceae as a function of the concentration of Silt. The line is a loess smoothing of the data, and the shaded area is the 95% confidence interval. (JPG 726 kb) [file 40168_2019_758_MOESM12_ESM.jpg]

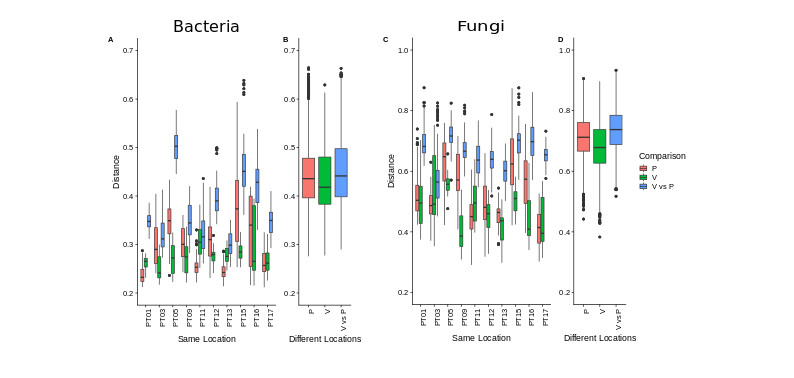

Supplement: Supplementary file 13 — Additional file 13: Figure S13. a) Boxplot of the Bray Curtis distances between the bacterial component of the microbiota of samples within the permanent grassland (P) and vineyard (V) soils and between vineyard and grassland soils (V vs P) for each site. b) same as a, across different sites. c) Same as a, for fungi. d) Same as b, for fungi. (JPG 47 kb) [file 40168_2019_758_MOESM13_ESM.jpg]
