# Supplementary material for: Microbiome of vineyard soils is shaped by geography and management
Source: Microbiome. 2019 Nov 8;7:140. doi: 10.1186/s40168-019-0758-7 (PMC6839268; doi:10.1186/s40168-019-0758-7)
Supplement: Supplementary file 14 — Additional file 14: Table S1. Main features of sample fields (A and B). All samples were from vineyards of Adige valley in Trentino region and sampled the very same day (July the 12th of 2017). Legend. V = vineyard, P1 = grassland at 8 m from V, P2 = grassland at 16 m from V (see also Additional file 1: Figure S1). Loc. Name = Location Name, Trellis sys. = Trellis system, m asl = meters at sea level. (DOCX 15 kb) [file 40168_2019_758_MOESM14_ESM.docx]

| **A)** | | | | |
| --- | --- | --- | --- | --- |
| **Code** | **Type** | **Loc. Name** | **Geographical Coordinates** | **Variety** |
| PT01 | V,P1,P2 | Besagno | 45°49'38.2"N 10°58'03.9"E | Chardonnay |
| PT03 | V,P1,P2 | Besagno | 45°49'49.1"N 10°58'54.5"E | Chardonnay |
| PT16 | V,P1,P2 | Besagno | 45°50'04.6"N 10°58'10.3"E | Chardonnay |
| PT05 | V,P1,P2 | Ala | 45°47'14.8"N 11°01'43.0"E | Chardonnay |
| PT09 | V,P1,P2 | Ala | 45°44'20.4"N 11°03'46.0"E | Muller Thurgau |
| PT12 | V,P1,P2 | Ala | 45°44'06.6"N 11°04'08.3"E | Muller Thurgau |
| PT15 | V,P1,P2 | Ala | 45°47'11.3"N 11°01'41.7"E | Chardonnay |
| PT11 | V,P1,P2 | Mori | 45°49'38.2"N 10°58'03.9"E | Chardonnay |
| PT17 | V,P1,P2 | Mori | 45°49'37.1"N 10°58'08.7"E | Chardonnay |
| PT13 | V,P1,P2 | S. Felice | 45°51'41.6"N 10°56'04.5"E | Pinot gris |

| **B)** | | | | |
| --- | --- | --- | --- | --- |
| **Code** | **Planting Year** | **Trellis Sys.** | **Altitude (m asl)** | **Previous crop** |
| PT01 | 1980 | pergola | 390 | vineyard |
| PT03 | 2009 | guyot | 489 | grassland |
| PT16 | 2008 | pergola | 420 | vineyard & grassland |
| PT05 | 1987 | pergola | 244 | vineyard |
| PT09 | 2001 | pergola | 660 | grassland |
| PT12 | 2004 | pergola | 677 | vineyard |
| PT15 | 2008 | pergola | 244 | grassland |
| PT11 | 2003 | guyot | 514 | grassland |
| PT13 | 2006 | pergola | 383 | arable |
| PT17 | 1986 | pergola | 514 | grassland |

**Additional file 14: Table S1.** Main features of sample fields (A and B). All samples were from vineyards of Adige valley in Trentino region and sampled the very same day ( July the 12th of 2017). Legend. V = vineyard, P1 = grassland at 8 m from V, P2 = grassland at 16 m from V (see also suppl. figure 21). Loc. Name = Location Name, Trellis sys. = Trellis system, m asl = meters at sea level.
