# Supplementary material for: Microbiome of vineyard soils is shaped by geography and management
Source: Microbiome. 2019 Nov 8;7:140. doi: 10.1186/s40168-019-0758-7 (PMC6839268; doi:10.1186/s40168-019-0758-7)
Supplement: Supplementary file 17 — Additional file 17: Table S4. The site-specific core microbiome for Bacteria and Archaea, and Fungi. (DOCX 14 kb) [file 40168_2019_758_MOESM17_ESM.docx]

| Site | # of Core OTUs (16S) | # of Core OTUs (ITS) |
| --- | --- | --- |
| PT01 Besagno | 549 | 68 |
| PT03 Besagno | 562 | 87 |
| PT16 Besagno | 422 | 43 |
| PT05 Ala | 396 | 32 |
| PT09 Ala | 554 | 31 |
| PT12 Ala | 536 | 43 |
| PT15 Ala | 372 | 43 |
| PT11 Mori | 638 | 70 |
| PT17 Mori | 599 | 63 |
| PT13 S. Felice | 581 | 76 |

**Additional file 17: Table S4.** The site-specific core microbiome for Bacteria and Archaea, and Fungi.
