# Supplementary material for: Microbiome of vineyard soils is shaped by geography and management
Source: Microbiome. 2019 Nov 8;7:140. doi: 10.1186/s40168-019-0758-7 (PMC6839268; doi:10.1186/s40168-019-0758-7)
Supplement: Supplementary file 18 — Additional file 18: Table S5. Pairwise comparison of location α-diversity measured by the Shannon Entropy for bacteria A) and fungi B) using the Wilcoxon rank-sum test, FDR corrected. Highlighted in bold values below the significance threshold of 0.05. (DOCX 14 kb) [file 40168_2019_758_MOESM18_ESM.docx]

| **A** | Ala | Besagno | Mori |
| --- | --- | --- | --- |
| Besagno | **0.00026** | - | - |
| Mori | 0.05099 | **7.3e-08** | - |
| S. Felice | **0.01470** | 0.83767 | **8.7e-05** |

| **B** | Ala | Besagno | Mori |
| --- | --- | --- | --- |
| Besagno | **0.0071** | - | - |
| Mori | 0.8474 | **0.0220** | - |
| S. Felice | **0.0306** | 0.8474 | **0.0354** |

**Additional file 18: Table S5.** Pairwise comparison of location α-diversity measured by the Shannon Entropy for bacteria **A**) and fungi **B**) using the Wilcoxon rank-sum test, FDR corrected. Highlighted in bold values below the significance threshold of 0.05.
