# Supplementary material for: Microbiome of vineyard soils is shaped by geography and management
Source: Microbiome. 2019 Nov 8;7:140. doi: 10.1186/s40168-019-0758-7 (PMC6839268; doi:10.1186/s40168-019-0758-7)
Supplement: Supplementary file 19 — Additional file 19: Table S6. Pairwise comparison of site α-diversity measured by the Shannon Entropy for bacteria a) and fungi b) using the Wilcoxon rank-sum test, FDR corrected. Comparisons are ordered by sampling area (PT13 is the only sample from S. Felice area). Highlighted in bold values below the significance threshold of 0.05 while light gray shade enlight comparisons inside the same area. (DOCX 17 kb) [file 40168_2019_758_MOESM19_ESM.docx]

| **A** | Besagno | | | Ala | | | | Mori | |
| --- | --- | --- | --- | --- | --- | --- | --- | --- | --- |
|  | PT01 | PT03 | PT16 | PT05 | PT09 | PT12 | PT15 | PT11 | PT17 |
| PT03 | 0.1652 |  |  |  |  |  |  |  |  |
| PT16 | **0.0001** | 0.8147 |  |  |  |  |  |  |  |
| PT05 | 0.4879 | 0.1897 | 0.4059 |  |  |  |  |  |  |
| PT09 | **2.1E-05** | **0.0001** | **6.9E-07** | **0.0001** |  |  |  |  |  |
| PT12 | 0.0684 | 0.3223 | **4.9E-06** | **0.0395** | 0.2327 |  |  |  |  |
| PT15 | 0.4254 | 0.8147 | **0.0003** | 0.2198 | **0.0039** | 0.2195 |  |  |  |
| PT11 | **4.5E-06** | **4.5E-06** | **1.2E-07** | **8.8E-06** | 0.0785 | 0.0918 | **8.8E-06** |  |  |
| PT17 | **0.0144** | 0.4659 | 7.1E-06 | 0.0915 | **8.3E-05** | 0.6651 | 0.4959 | **4.0E-06** |  |
| PT13 | 0.3059 | 0.0785 | 0.1716 | 0.6070 | **5.1E-05** | **0.0144** | 0.1716 | **2.3E-06** | **0.0161** |

| **B** | Besagno | | | Ala | | | | Mori | |
| --- | --- | --- | --- | --- | --- | --- | --- | --- | --- |
|  | PT01 | PT03 | PT16 | PT05 | PT09 | PT12 | PT15 | P11 | PT17 |
| PT03 | 0.0592 |  |  |  |  |  |  |  |  |
| PT16 | **0.0024** | 0.055 |  |  |  |  |  |  |  |
| PT05 | **0.0097** | 0.1379 | 0.5039 |  |  |  |  |  |  |
| PT09 | **0.0024** | **0.0056** | 0.5039 | 0.8547 |  |  |  |  |  |
| PT12 | **0.0024** | **0.0055** | 0.9337 | 0.5414 | 0.3580 |  |  |  |  |
| PT15 | **0.0055** | 0.0746 | 0.2527 | 0.6804 | 0.6339 | 0.1176 |  |  |  |
| PT11 | **0.0065** | 0.0665 | 0.1524 | 0.6692 | 0.5039 | 0.0980 | 0.8470 |  |  |
| PT17 | **0.0024** | **0.0055** | 1.0000 | 0.5527 | 0.5039 | 0.8413 | 0.1524 | 0.1524 |  |
| PT13 | **0.0352** | 0.6416 | **0.0363** | 0.3343 | **0.0352** | **0.0266** | 0.3080 | 0.2527 | **0.0352** |

**Additional file 19: Table S6.** Pairwise comparison of site α-diversity measured by the Shannon Entropy for bacteria a) and fungi b) using the Wilcoxon rank-sum test, FDR corrected. Comparisons are ordered by sampling area (PT13 is the only sample from S. Felice area). Highlighted in bold values below the significance threshold of 0.05 while light grey shade enlight comparisons inside the same area.
