# Supplementary material for: Microbiome of vineyard soils is shaped by geography and management
Source: Microbiome. 2019 Nov 8;7:140. doi: 10.1186/s40168-019-0758-7 (PMC6839268; doi:10.1186/s40168-019-0758-7)
Supplement: Supplementary file 20 — Additional file 20: Table S7. Average differences of the α-diversity of bacterial and fungal communities measured by the Shannon entropy between the Vineyards and P1 samples for each site. Statistically significant contrasts are highlighted in bold. While for bacteria in no case the P1 and P2 samples have significantly different richness, for fungi, in two cases the α-diversity of the P2 samples are significantly different from the P1 samples (higher in PT16 and lower in PT17, p-values 0.00116 and 0.040, respectively). (DOCX 14 kb) [file 40168_2019_758_MOESM20_ESM.docx]

| Site | Bacteria | Fungi |
| --- | --- | --- |
| **PT01** | **0.216** | **-0.410** |
| PT03 | 0.0326 | -0.237 |
| **PT05** | **-0.472** | **-0.495** |
| PT09 | 0.077 | 0.138 |
| PT11 | -0.075 | 0.125 |
| **PT12** | **0.385** | 0.231 |
| **PT13** | **0.293** | **-0.361** |
| PT15 | 0.094 | **0.322** |
| PT16 | 0.112 | 0.082 |
| PT17 | 0.033 | -0.0012 |

**Additional file 20: Table S7.** Average differences of the α-diversity of bacterial and fungal communities measured by the Shannon entropy between the Vineyards and P1 samples for each site. Statistically significant contrasts are highlighted in bold. While for bacteria in no case the P1 and P2 samples have significantly different richness, for fungi, in two cases the α-diversity of the P2 samples are significantly different from the P1 samples (higher in PT16 and lower in PT17, p-values 0.00116 and 0.040, respectively)
