# Supplementary material for: Microbiome of vineyard soils is shaped by geography and management
Source: Microbiome. 2019 Nov 8;7:140. doi: 10.1186/s40168-019-0758-7 (PMC6839268; doi:10.1186/s40168-019-0758-7)
Supplement: Supplementary file 21 — Additional file 21: Table S8. Linear model correlating the the bacterial and fungal α-diversities in all sites. (DOCX 14 kb) [file 40168_2019_758_MOESM21_ESM.docx]

##

## Call:

## lm(formula = Shannon_Fungi ~ Shannon_Bacteria, data=Shannon)

##

## Residuals:

## Min 1Q Median 3Q Max

## -1.22041 -0.17531 0.01465 0.21689 0.73534

##

## Coefficients:

## Estimate Std. Error t value Pr(>|t|)

## (Intercept) 4.65958 0.79046 5.895 1.9e-08 ***

## Shannon_Bacteria -0.02132 0.12268 -0.174 0.862

## ---

## Signif. codes: 0 '***' 0.001 '**' 0.01 '*' 0.05 '.' 0.1 ' ' 1

##

## Residual standard error: 0.3171 on 174 degrees of freedom

## Multiple R-squared: 0.0001735, Adjusted R-squared: -0.005573

## F-statistic: 0.0302 on 1 and 174 DF, p-value: 0.8622

**Additional file 21: TableS8.** Linear model correlating the the bacterial and fungal α-diversities in all sites
