# Supplementary material for: Microbiome of vineyard soils is shaped by geography and management
Source: Microbiome. 2019 Nov 8;7:140. doi: 10.1186/s40168-019-0758-7 (PMC6839268; doi:10.1186/s40168-019-0758-7)
Supplement: Supplementary file 22 — Additional file 22: Table S9. Linear model correlating the the bacterial and fungal α-diversities, stratified by site. (DOCX 14 kb) [file 40168_2019_758_MOESM22_ESM.docx]

##

## Call:

## lm(formula = Shannon_Fungi ~ Shannon_Bacteria + Shannon_Bacteria *

## Site, data = Shannon)

##

## Residuals:

## Min 1Q Median 3Q Max

## -1.59690 -0.08195 0.02813 0.16380 0.58100

##

## Coefficients:

## Estimate Std. Error t value Pr(>|t|)

## (Intercept) 16.2267 3.7602 4.315 2.82e-05 ***

## Shannon_Bacteria -1.7904 0.5880 -3.045 0.002735 **

## SitePT03 -10.2099 5.5963 -1.824 0.070002 .

## SitePT05 -17.4945 4.1814 -4.184 4.77e-05 ***

## SitePT09 -6.9768 6.8416 -1.020 0.309420

## SitePT11 -5.7608 6.1522 -0.936 0.350529

## SitePT12 -16.0202 4.3661 -3.669 0.000333 ***

## SitePT13 -6.0898 4.5167 -1.348 0.179522

## SitePT15 -10.2498 4.4815 -2.287 0.023531 *

## SitePT16 -8.0529 4.7833 -1.684 0.094266 .

## SitePT17 -1.2643 8.3337 -0.152 0.879611

## Shannon_Bacteria:SitePT03 1.5832 0.8710 1.818 0.071033 .

## Shannon_Bacteria:SitePT05 2.7030 0.6558 4.121 6.10e-05 ***

## Shannon_Bacteria:SitePT09 1.0661 1.0469 1.018 0.310072

## Shannon_Bacteria:SitePT11 0.8988 0.9388 0.957 0.339844

## Shannon_Bacteria:SitePT12 2.4332 0.6789 3.584 0.000452 ***

## Shannon_Bacteria:SitePT13 0.9217 0.7080 1.302 0.194920

## Shannon_Bacteria:SitePT15 1.5597 0.6996 2.229 0.027227 *

## Shannon_Bacteria:SitePT16 1.1747 0.7569 1.552 0.122690

## Shannon_Bacteria:SitePT17 0.1601 1.2903 0.124 0.901393

## ---

## Signif. codes: 0 '***' 0.001 '**' 0.01 '*' 0.05 '.' 0.1 ' ' 1

##

## Residual standard error: 0.2776 on 156 degrees of freedom

## Multiple R-squared: 0.3126, Adjusted R-squared: 0.2288

## F-statistic: 3.733 on 19 and 156 DF, p-value: 2.219e-06

Additional file 22: Table S9. Linear model correlating the the bacterial and fungal α-diversities, stratified by site
