# Supplementary material for: Microbiome of vineyard soils is shaped by geography and management
Source: Microbiome. 2019 Nov 8;7:140. doi: 10.1186/s40168-019-0758-7 (PMC6839268; doi:10.1186/s40168-019-0758-7)
Supplement: Supplementary file 23 — Additional file 23: Table S10. Linear model correlating the the bacterial and fungal α-diversities for PT05. (DOCX 13 kb) [file 40168_2019_758_MOESM23_ESM.docx]

##

## Call:

## lm(formula = Shannon_Fungi ~ Shannon_Bacteria, data = Shannon_PT05)

##

## Residuals:

## Min 1Q Median 3Q Max

## -0.49217 -0.10500 0.03302 0.12950 0.28935

##

## Coefficients:

## Estimate Std. Error t value Pr(>|t|)

## (Intercept) -1.2678 1.4504 -0.874 0.39790

## Shannon_Bacteria 0.9126 0.2303 3.963 0.00162 **

## ---

## Signif. codes: 0 '***' 0.001 '**' 0.01 '*' 0.05 '.' 0.1 ' ' 1

##

## Residual standard error: 0.2202 on 13 degrees of freedom

## Multiple R-squared: 0.5471, Adjusted R-squared: 0.5123

## F-statistic: 15.71 on 1 and 13 DF, p-value: 0.001621

**Additional file 23: Table S10** Linear model correlating the the bacterial and fungal α-diversities for PT05
