# Supplementary material for: Microbiome of vineyard soils is shaped by geography and management
Source: Microbiome. 2019 Nov 8;7:140. doi: 10.1186/s40168-019-0758-7 (PMC6839268; doi:10.1186/s40168-019-0758-7)
Supplement: Supplementary file 24 — Additional file 24: Table S11. Linear model correlating the the bacterial and fungal α-diversities for PT12. (DOCX 13 kb) [file 40168_2019_758_MOESM24_ESM.docx]

##

## Call:

## lm(formula = Shannon_Fungi ~ Shannon_Bacteria, data = Shannon_PT12)

##

## Residuals:

## Min 1Q Median 3Q Max

## -0.29869 -0.07578 -0.03047 0.08326 0.18265

##

## Coefficients:

## Estimate Std. Error t value Pr(>|t|)

## (Intercept) 0.2065 1.0230 0.202 0.84258

## Shannon_Bacteria 0.6428 0.1564 4.109 0.00082 ***

## ---

## Signif. codes: 0 '***' 0.001 '**' 0.01 '*' 0.05 '.' 0.1 ' ' 1

##

## Residual standard error: 0.128 on 16 degrees of freedom

## Multiple R-squared: 0.5135, Adjusted R-squared: 0.4831

## F-statistic: 16.89 on 1 and 16 DF, p-value: 0.0008204

**Additional file 24: Table S11** Linear model correlating the the bacterial and fungal α-diversities for PT12
