# Supplementary material for: Microbiome of vineyard soils is shaped by geography and management
Source: Microbiome. 2019 Nov 8;7:140. doi: 10.1186/s40168-019-0758-7 (PMC6839268; doi:10.1186/s40168-019-0758-7)
Supplement: Supplementary file 25 — Additional file 25: Table S12. Linear mixed effect model correlating the the bacterial and fungal α-diversities for all sites except PT05 and PT12. Intercept is a treated as a random effect, slope as a fixed effect. (DOCX 13 kb) [file 40168_2019_758_MOESM25_ESM.docx]

## Linear mixed-effects model fit by REML

## Data: Shannon_other

## AIC BIC logLik

## 82.34327 94.13831 -37.17164

##

## Random effects:

## Formula: ~1 | Site

## (Intercept) Residual

## StdDev: 0.1576361 0.2949162

##

## Fixed effects: Shannon_Fungi ~ Shannon_Bacteria

## Value Std.Error DF t-value p-value

## (Intercept) 8.341658 1.2041873 134 6.927210 0.000

## Shannon_Bacteria -0.589575 0.1866508 134 -3.158704 0.002

## Correlation:

## (Intr)

## Shannon_Bacteria -0.999

##

## Standardized Within-Group Residuals:

## Min Q1 Med Q3 Max

## -5.01891462 -0.31560830 0.08332005 0.59582981 1.83970082

##

## Number of Observations: 143

## Number of Groups: 8

**Additional file 25: Table S12** Linear mixed effect model correlating the the bacterial and fungal α-diversities for all sites except PT05 and PT12. Intercept is a treated as a random effect, slope as a fixed effect.
