# Supplementary material for: Microbiome of vineyard soils is shaped by geography and management
Source: Microbiome. 2019 Nov 8;7:140. doi: 10.1186/s40168-019-0758-7 (PMC6839268; doi:10.1186/s40168-019-0758-7)
Supplement: Supplementary file 26 — Additional file 26: Table S13. Parameters of the linear models in Additional file 6: Figure S6 modeling the richness of bacterial microbiota (Shannon entropy) against the chemical characteristics of the soil. (DOCX 14 kb) [file 40168_2019_758_MOESM26_ESM.docx]

|  | **Intercept** | | **Slope** | | **Adjusted R^2^** | **p-value** |
| --- | --- | --- | --- | --- | --- | --- |
|  | **Value** | **p-value** | **Value** | **p-value** |  |  |
| **CaCO_3_** | 6.360 | < 2e-16 | 4.561e-04 | 2.01e-07 | 0.1416 | 2.011e-07 |
| **Sand** | 6.9048854 | < 2e-16 | -0.0011854 | 5.72e-16 | 0.3151 | 5.716e-16 |
| **Silt** | 5.8437884 | <2e-16 | 0.0012862 | <2e-16 | 0.3649 | < 2.2e-16 |
| **Zn** | 6.476715 | <2e-16 | -0.004968 | 0.0185 | 0.02634 | 0.01853 |
| **Cu** | 6.5003705 | < 2e-16 | -0.0025483 | 4.34e-07 | 0.134 | 4.345e-07 |
| **pH** | 6.35450 | <2e-16 | 0.01174 | 0.808 | -0.0055 | 0.8081 |

**Additional file 26: Table S13**  Parameters of the linear models in Supplementary Figure 6 modelling the richness of bacterial microbiota (Shannon entropy) against the chemical characteristics of the soil.
