# Supplementary material for: Microbiome of vineyard soils is shaped by geography and management
Source: Microbiome. 2019 Nov 8;7:140. doi: 10.1186/s40168-019-0758-7 (PMC6839268; doi:10.1186/s40168-019-0758-7)
Supplement: Supplementary file 27 — Additional file 27: Table S14. Parameters of the linear models in Additional file 7: Figure S7 modeling the richness of fungal microbiota (Shannon entropy) against the chemical characteristics of the soil. (DOCX 14 kb) [file 40168_2019_758_MOESM27_ESM.docx]

|  | **Intercept** | | **Slope** | | **Adjusted R^2^** | **p-value** |
| --- | --- | --- | --- | --- | --- | --- |
|  | **Value** | **p-value** | **Value** | **p-value** |  |  |
| **Cu** | 4.5917273 | < 2e-16 | -0.0026240 | 0.00173 | 0.05012 | 0.001733 |
| **Silt** | 4.576 | <2e-16 | -9.883e-05 | 0.708 | -0.004989 | 0.7076 |
| **Zn** | 4.598249 | < 2e-16 | -0.009362 | 0.00616 | 0.03723 | 0.006163 |
| **N** | 4.59887 | <2e-16 | -0.02035 | 0.237 | 0.002366 | 0.2366 |
| **Organic** | 4.6013368 | <2e-16 | -0.0011746 | 0.233 | 0.002487 | 0.2332 |
| **Sand** | 4.3960080 | <2e-16 | 0.0003434 | 0.187 | 0.00436 | 0.1867 |

**Additional file 27: Table S14**  Parameters of the linear models in Supplementary Figure 7 modelling the richness of fungal microbiota (Shannon entropy) against the chemical characteristics of the soil.
