# Supplementary material for: Microbiome of vineyard soils is shaped by geography and management
Source: Microbiome. 2019 Nov 8;7:140. doi: 10.1186/s40168-019-0758-7 (PMC6839268; doi:10.1186/s40168-019-0758-7)
Supplement: Supplementary file 29 — Additional file 29: Table S16. PERMANOVA results for the distinction between P1 and P2 (left) and P1 + P2 and V (right) for bacteria and fungi. In the P1 + P2 vs V comparison the P1 and P2 samples were considered together. Significant p-values (p < 0.01) are marked in bold. (DOCX 14 kb) [file 40168_2019_758_MOESM29_ESM.docx]

| **Site** | **Bacteria** | | **Fungi** | |
| --- | --- | --- | --- | --- |
|  | **P1 vs P2** | **P1+P2 vs V** | **P1 vs P2** | **P1+P2 vs V** |
| PT01 | 0.05 | **0.0001** | **0.004** | **0.0001** |
| PT03 | 0.19 | **0.0005** | 0.04 | **0.0001** |
| PT05 | 0.01 | **0.0003** | 0.02 | **0.0005** |
| PT09 | 0.12 | **0.0003** | 0.04 | **0.0001** |
| PT11 | 0.50 | **0.0001** | 0.07 | **0.0002** |
| PT12 | 0.13 | **0.0002** | 0.02 | **0.0001** |
| PT13 | 0.06 | **0.0003** | 0.03 | **0.0002** |
| PT15 | 0.05 | **0.0002** | 0.04 | **0.0002** |
| PT16 | **0.002** | **0.0001** | **0.001** | **0.0001** |
| PT17 | 0.01 | **0.0002** | 0.01 | **0.0001** |

**Additional file 29: Table S16.** PERMANOVA results for the distinction between P1 and P2 (left) and P1+P2 and V (right)for bacteria and fungi. In the P1+P2 vs V comparison the P1 and P2 samples were considered together. Significant p-values (p<0.01) are marked in bold.
